# Put the CAR-T before the HRS: Advances in Anti-CD30 Immunotherapy Targeting Hodgkin/Reed-Sternberg Cells in Classical Hodgkin Lymphoma

**DOI:** 10.32604/or.2025.073008

**Published:** 2026-02-24

**Authors:** Yuriy Mayasin, Maria Osinnikova, Daria Osadchaya, Victoria Dmitrienko, Anna Gorodilova, Chulpan Kharisova, Kristina Kitaeva, Valeria Solovyeva, Albert Rizvanov

**Affiliations:** 1 Institute of Fundamental Medicine and Biology, Kazan Federal University, Kazan, 420008, Russia; 2 Translational Medicine Research Center, Sirius University of Science and Technology, Sirius, 354340, Russia; 3 Division of Medical and Biological Sciences, Tatarstan Academy of Sciences, Kazan, 420111, Russia

**Keywords:** Hodgkin lymphoma, chimeric antigen receptor T cells, immunotherapy, monoclonal antibodies, Hodgkin/reed-Sternberg cells, clinical trials

## Abstract

Classical Hodgkin lymphoma (cHL) is characterized by rare Hodgkin/Reed-Sternberg (HRS) tumor cells that uniformly express cluster of differentiation (CD)30 molecules and orchestrate an immunosuppressive tumor microenvironment, making CD30 an attractive and selective therapeutic target. We summarize the biological rationale for CD30 as a therapeutic target and the preclinical and clinical evidence across major platforms: antibody-drug conjugates (brentuximab vedotin), monoclonal antibodies (including acimtamig and its combinations with Natural Killer cells), second- and third-generation chimeric antigen receptor (CAR)-T cells, and alternative modalities. Particular attention is given to standardized response assessment (IWG, Lugano, RECIL criteria), which enables appropriate cross-trial comparisons. Taken together, the data indicate that beyond the established role of brentuximab vedotin, CD30-directed CAR-T cells and bispecific antibodies demonstrate high activity in refractory cHL, especially when used with fludarabine-containing lymphodepletion, combined with programmed cell death 1 (PD-1) receptor blockade as a strategy to eradicate minimal residual disease. Key challenges include durable effector-cell persistence and optimization of sequencing and combinations; notably, loss of CD30 as an escape mechanism appears uncommon. Integrating mechanistic insights into HRS biology with clinical trial data highlights strategies to enhance the efficacy, safety, and accessibility of CD30-directed immunotherapy. This review aims to provide a concise overview of CD30-targeted approaches in cHL, emphasizing therapeutic outcomes and the evolution of CAR-T technologies.

## 1 Introduction

Hodgkin lymphoma (HL) is a malignant B cell neoplasm mostly defined by the presence of Hodgkin and Reed–Sternberg (HRS) tumor cells, which originate from germinal center B cells but downregulate canonical B cell markers, such as CD19, and instead stably express CD15 and CD30 [1–3]. HRS cells comprise about 1% of the tumor cellularity, yet they actively remodel the tumor microenvironment (TME), recruiting immune cells and creating an immunosuppressive milieu that promotes tumor survival [4,5]. Epstein–Barr virus (EBV) is associated with approximately half of cHL cases and drives activation of signaling pathways including Nuclear Factor kappa-light-chain-enhancer of activated B cells (NF-κB) and Janus kinase–signal transducer and activator of transcription (JAK–STAT), as well as upregulation of programmed death-ligand 1/2 (PD-L1/PD-L2), thereby enhancing immune evasion and resistance to apoptosis [6].

CD30, a cell-surface receptor of the tumor necrosis factor receptor superfamily, is highly expressed on HRS cells but expressed at low levels in normal tissues; it engages NF-κB and mitogen-activated protein kinase (MAPK) signaling, promoting tumor-cell survival [7]. Although most patients achieve durable remission with standard therapy, up to 30% develop relapsed/refractory (R/R) disease, underscoring the need for more selective and effective approaches [8]. The emergence of immunotherapies—such as antibody-drug conjugates (ADCs), antibodies and chimeric antigen receptor (CAR) T-cell therapy targeting CD30 offers new treatment options. Their efficacy has been demonstrated in preclinical and clinical studies, which are reviewed in this paper [9].

The aim of this review is to systematize and analyze both contemporary and retrospective data on CD30-directed immunotherapy for classical Hodgkin lymphoma, including the evaluation of therapeutic efficacy in preclinical and clinical studies with particular emphasis on response-assessment criteria, as well as to provide a detailed examination of the evolution of CAR-T technology generations and their application in combination with lymphodepleting regimens.

## 2 Hodgkin Lymphoma and Hodgkin/Reed-Sternberg Cells

Hodgkin lymphoma is a rare neoplasm of the lymphatic system and among the most common malignancies in young adults [10]. The disease is characterized by CD30-positive HRS tumor cells, encompassing mononuclear Hodgkin cells and giant multinucleated Reed-Sternberg (RS) cells, which derive from germinal center B cells [11,12]. In 1898, Carl Sternberg described large multinucleated cells in lymph nodes from cases he interpreted as a form of tuberculosis of the lymphatic apparatus presenting as pseudoleukemia [13]. Independently, in 1902 Dorothy Reed described these multinucleated cells and argued that they defined a distinct disease entity—Hodgkin’s disease (now termed Hodgkin lymphoma), rather than tuberculosis [14,15]. She also noted a population of single large tumor cells with a single nucleus and prominent nucleolus, later termed Hodgkin cells [16]. A prevailing hypothesis posits that Hodgkin cells can give rise to RS cells via incomplete cytokinesis and subsequent fusion of daughter cells [17,18]. The combined population, later termed HRS cells, has become one of the defining hallmarks of Hodgkin lymphoma.

An important etiologic factor is EBV, detected in approximately 45% of HL cases [19]. However, in some subtypes, such as nodular sclerosis HL, the intracellular EBV genome is infrequently detected [20]. Human immunodeficiency virus (HIV)-positive individuals also have an increased risk of developing HL. In the era of highly active antiretroviral therapy (HAART), the incidence of HIV-associated HL has paradoxically increased; notably, risk appears lower with severe immunosuppression than with moderate immunosuppression [21,22]. Beyond EBV and HIV infections, the etiologic framework of cHL additionally encompasses germline susceptibility centered on HLA class II with additional non-HLA loci (e.g., REL at 2p16.1, 8q24.21, GATA3 at 10p14) [23] and iatrogenic or primary immunosuppression, notably after solid-organ or hematopoietic transplantation and in some autoimmune diseases [24,25].

EBV-mediated activation of NF-κB and JAK–STAT signaling, together with upregulation of PD-L1/PD-L2, confers increased resistance to apoptosis in HRS cells [26]. In HRS cells, EBV typically exhibits latency type II, characterized by the expression of the viral genes EBNA1, LMP1, and LMP2A, as well as pull of non-coding RNAs [27]. The latent LMP1 mimics a constitutively active CD40 receptor, recruiting TRAFs and activating canonical and non-canonical NF-κB signaling, which induces anti-apoptotic proteins such as BCL-2 and BCL-XL and drives cytokine secretion [4,28]. LMP2A, through ITAMs, activates PI3K/Akt and ERK/MAPK pathways, delivering a tonic “B-cell receptor-like” survival signal and further promoting HRS cell resilience [29]. EBV^+^ HRS cells also show enhanced JAK2/STAT3 activation, sometimes compounded by inactivating mutations in negative regulators such as SOCS1 [30].

Beyond its intrinsic signaling effects, EBV profoundly remodels the tumor microenvironment. LMP1- and EBER-driven secretion of interleukin (IL)-10, C-C motif chemokine ligand (CCL)20, and transforming growth factor beta (TGF-β) attracts regulatory T-cells and M2-polarized macrophages, while upregulation of PD-L1 on HRS cells and surrounding myeloid cells dampens cytotoxic T-cell activity [31]. EBV-associated epigenetic modifications, including DNA methylation and chromatin remodeling, further stabilize the malignant phenotype [32]. Collectively, these viral mechanisms sustain HRS-cell survival and immune evasion, foster a suppressive TME, and may underlie the distinct clinical and molecular features observed in EBV^+^ cHL.

In addition, HL-specific amplification of the 9p24.1 locus leads to PD-L1 and PD-L2 overexpression on HRS cells, promoting immune evasion and tumor progression [33,34]. Although few in number, HRS cells secrete large pools of cytokines and chemokines, including CCL5, CCL17, CCL22, and IL-7, which recruit T helper type 2 (Th2) cells, regulatory T-cells (Tregs), macrophages, and eosinophils, thereby extensively remodeling the TME of the lymph node [35–37]. These recruited cells create an immunosuppressive niche and support tumor growth by delivering survival signals [38]. Immune evasion is further mediated by loss of major histocompatibility complex (MHC) class I expression, secretion of IL-10 and TGF-β, and upregulation of Fas ligand (FasL) on HRS cells, with PD-L1/PD-L2 engaging programmed cell death protein 1 (PD-1) on T-cells and promoting T-cell exhaustion [39–41].

### 2.1 Classification of Hodgkin Lymphoma

Approximately 83,000 new cases of HL are reported worldwide each year. HL most commonly affects young adults (15–35 years) and older adults (≥55 years), with the incidence in men about 1.5 times that in women [42]. The World Health Organization (WHO) projects an incidence of 85,000–90,000 new HL cases annually in 2024–2025. In the European Union, about 25,000 new cases are reported each year, with the highest incidence in Northern and Western Europe (e.g., the United Kingdom, Germany, Sweden). In the United States, 8570 new HL diagnoses occur annually [43]; for 2025, 8720 new cases and 1150 deaths are projected [44]. In many Asian countries, HL incidence is lower than in Europe and the United States; subtype distributions may also differ, with reports from South and Southeast Asia describing distinct patterns [45].

The WHO classification divides HL into two main categories based on morphologic and immunohistochemical characteristics: classical Hodgkin lymphoma (cHL) and nodular lymphocyte-predominant Hodgkin lymphoma (NLPHL). cHL is a group of B cell neoplasms derived from germinal center B cells and characterized by a small number of tumor cells embedded in a reactive microenvironment rich in immune cells [46]. The presence of malignant HRS cells is a hallmark of cHL; these cells are consistently found across subtypes and are positive for CD15 in up to 85% of cases, uniformly positive for CD30, and essentially negative for CD45 on immunohistochemical staining [47,48]. cHL is divided into four subtypes: nodular sclerosis, mixed cellularity, lymphocyte-rich, and lymphocyte-depleted.

Nodular sclerosis cHL (NS-cHL) is the most common subtype and is associated with a more favorable prognosis than other subtypes [49]. It accounts for approximately 70% of cHL cases and is characterized by lacunar-type RS cells and broad sclerosing bands within an inflammatory background [50]. Mixed cellularity cHL (MC-cHL) accounts for between 20% and 25% of cHL cases in the United States and is associated with a less favorable prognosis [49]. It features a diffuse, mixed inflammatory background without sclerosis and is frequently associated with EBV and HIV infection [51]. Lymphocyte-rich cHL (LR-cHL) shows a nodular or diffuse background of small lymphocytes without neutrophils or eosinophils. It represents about 5% of cHL cases, typically presents at an early stage, rarely forms bulky disease, and carries an excellent prognosis with contemporary therapy [52,53]. Lymphocyte-depleted cHL (LD-cHL) is rare disease (<1% of cases), usually exhibits aggressive clinical behavior, and is associated with an unfavorable prognosis [54]. It is characterized by diffuse proliferation of HRS cells with a markedly scant inflammatory background [55]. NLPHL is a rare, indolent lymphoma that represents a distinct category of HL [46]. Unlike cHL, the neoplastic cells in NLPHL express B cell antigens such as CD20 and lack CD30 [56].

The heterogeneity of subtypes, associations with EBV and HIV, and the persistence of R/R disease despite standard therapy underscore the need to develop targeted immunotherapeutic approaches directed at key molecular targets and mechanisms of immune evasion.

### 2.2 Standard Treatment Approaches in cHL

Curative therapy for cHL has traditionally combined multi-agent chemotherapy with involved-site radiotherapy (ISRT). In limited-stage disease, two to four cycles of doxorubicin, bleomycin, vinblastine, and dacarbazine (ABVD) followed by ISRT remain a common standard, and positron emission tomography (PET)–adapted strategies allow de-escalation, for example omission of bleomycin after a negative interim PET, which reduces pulmonary toxicity without loss of efficacy [57,58]. In advanced stages, ABVD is widely used; risk-adapted intensification with escalated bleomycin, etoposide, doxorubicin, cyclophosphamide, vincristine, procarbazine, and prednisone (escalated BEACOPP) or the brentuximab vedotin (BV)-containing BrECADD regimen can improve progression-free control in selected patients, although this comes with higher rates of myelosuppression, gonadal damage, and other late effects, so many groups employ PET-guided algorithms to balance efficacy and toxicity [59].

Despite high initial cure rates, approximately 20 to 30 percent of patients develop R/R cHL, particularly those with advanced-stage or bulky disease. The standard approach to R/R cHL uses platinum-based salvage chemotherapy such as ifosfamide, carboplatin, and etoposide (ICE), dexamethasone, high-dose cytarabine, and cisplatin (DHAP), or gemcitabine, dexamethasone, and cisplatin (GDP), followed by high-dose chemotherapy and autologous stem-cell transplantation (ASCT) for chemosensitive relapse. Consolidation with the anti-CD30 antibody-drug conjugate BV after ASCT improves PFS in high-risk patients, as demonstrated in the AETHERA trial [60]. Nevertheless, outcomes remain suboptimal after relapse post-ASCT when only conventional cytotoxic therapy is available, and cumulative late toxicities from anthracyclines, alkylators, bleomycin, and mediastinal radiotherapy, including cardiopulmonary injury, infertility, and second malignant neoplasms, are well documented [61,62].

Recent randomized data show that nivolumab plus doxorubicin, vinblastine, and dacarbazine (nivolumab-AVD) yields longer PFS and a more favorable safety profile than BV plus AVD in stage III to IV cHL, and contemporary guidelines increasingly reflect these options for frontline therapy selection. These developments highlight a shift toward mechanism-based strategies and provide a natural bridge to the focus of the main article, namely targeted immunotherapy for R/R cHL [63].

### 2.3 Tumor Microenvironment of cHL

HRS cells orchestrate a highly heterogeneous cHL TME composed of innate (eosinophils, neutrophils, mast cells, macrophages, NK cells) and adaptive immune cells (non-neoplastic B cells, plasma cells, CD4^+^ T helper cells, Tregs and CD8^+^ T-cells), as well as stromal cells and fibroblasts [64].

Within the innate immune compartment, macrophages constitute an important yet highly plastic population whose pro- or antitumor effects are largely dictated by signals from the TME [65,66]. In cHL, most studies report a predominance of pro-tumor (immunosuppressive, often M2-polarized) macrophage states [38,67]. Tumor-associated macrophages (TAM), which are predominantly PD-L1^+^, form dense clusters adjacent to HRS cells [68]. Driven by IL-10, TGF-β, and CCL5—C-C chemokine receptor (CCR)5 signaling, they acquire an M2-like polarization [69]. In this state, TAMs secrete IL-10, vascular endothelial growth Factor (VEGF) and other anti-inflammatory mediators that promote angiogenesis and stromal remodeling [70]. High TAM density consistently correlates with poor prognosis and reduced survival in cHL [71]. Eosinophils are recruited largely by CCL5 and IL-5 [72]; they express CD30L, whose engagement of CD30 on HRS cells augments proliferation [73], and they produce TGF-β, contributing to fibrotic stroma, particularly in nodular sclerosis [74]. Neutrophils are recruited mainly by C-X-C motif chemokine ligand (CXCL)8/IL-8 [75]; they produce a proliferation-inducing ligand (APRIL), activating B cell maturation antigen (BCMA) and transmembrane activator and CAML interactor (TACI) on HRS cells, and secrete nerve growth factor (NGF), which stimulates tropomyosin receptor kinase A (TRKA) signaling and supports tumor-cell survival [76,77]. Neutrophils also generate neutrophil extracellular traps (NETs), reactive oxygen species (ROS), arginase-1 and metalloproteinases (MMPs), thereby enhancing immunosuppression and tumor invasion [78–80]. Mast cells localize preferentially to fibrotic areas in nodular sclerosis [81]; they are activated by chemokines including CCL5 and by IL-9 [37,82], express CD30L, and signal to HRS cells via the CD30/CD30L axis [83]. In addition, mast cells secrete VEGF, IL-13 and TGF-β, promoting angiogenesis, fibrosis and extracellular matrix (ECM) remodeling [84]. NK cells, which recognize low MHC class I on HRS cells, are functionally suppressed via PD-1/PD-L1 interactions, TGF-β, and Human Leukocyte Antigen (HLA)-G/E expression on tumor cells, leading to diminished cytotoxicity [85,86]. Dendritic cells (DCs), including CCR6^+^ subsets, are attracted by CCL20 [4,87] but exhibit impaired migration to lymphoid organs and reduced antigen-presenting function in cHL [88,89]. HRS cells further reprogram DCs through IL-10 and Migration Inhibitory Factor (MIF) and engage Hepatocyte Growth Factor (HGF)/Mesenchymal-Epithelial Transition factor (c-MET) axis to promote their own survival [90,91].

**Figure 1:**
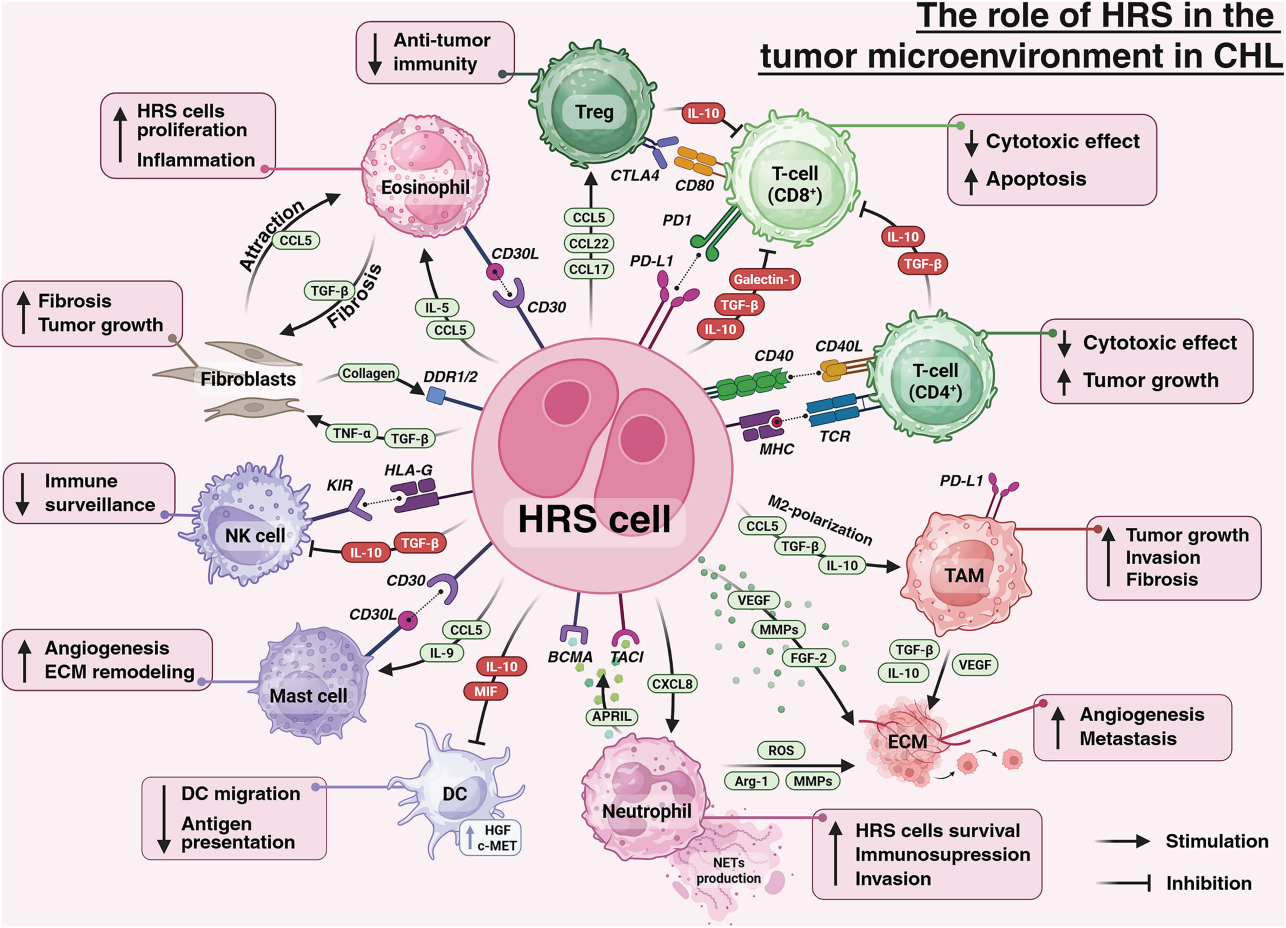
The role of HRS cells in tumor microenvironment in classic Hodgkin Lymphoma. Abbreviations: APRIL—a proliferation-inducing ligand, Arg-1—arginase-1, BCMA—B cell maturation antigen, CCL—C-C motif chemokine ligand, CD—cluster of differentiation, c-MET—MET receptor tyrosine kinase, CTLA-4—cytotoxic T-lymphocyte–associated protein 4, CXCL—C-X-C motif chemokine ligand, DC—dendritic cell, DDR—discoidin domain receptor, ECM—extracellular matrix, FGF—fibroblast growth factor, HGF—hepatocyte growth factor, HLA-G—human leukocyte antigen G, HRS—Hodgkin and Reed–Sternberg cells, IL—interleukin, KIR—killer-cell immunoglobulin-like receptor, MHC—major histocompatibility complex, MIF—macrophage migration inhibitory factor, MMPs—matrix metalloproteinases, NETs—neutrophil extracellular traps, NK—natural killer, PD1—programmed cell death protein 1, PD-1L—programmed death-ligand 1, ROS—reactive oxygen species, TACI—transmembrane activator and CAML interactor, TAM—tumor-associated macrophages, TCR—T-cell receptor, TGF-β—transforming growth factor beta, TNF-α—tumor necrosis factor alpha, Treg—regulatory T cell, VEGF—vascular endothelial growth factor. Created using BioRender. Mayasin, Y. (2025), https://BioRender.com/6ges05j (accessed on 07 August 2025)

Within the adaptive compartment, CD4^+^ T-cells form characteristic rosettes around HRS cells, providing contact-dependent signals via CD40L–CD40 and CD54–CD11a interactions [92–94]. They secrete IL-10 and TGF-β, yet their effector function is attenuated owing to chronic PD-1 signaling and other exhaustion mechanisms [68,95]. CD8^+^ T-cells likewise display features of exhaustion in the TME [96]. HRS cells can express FasL, inducing apoptosis of CD95^+^ cytotoxic T-cells and further diminishing antitumor activity [97]. Tregs are recruited predominantly via the CCL17/CCL22–CCR4 axis [35,98]; they secrete IL-10 and TGF-β and express Cytotoxic T-Lymphocyte Antigen 4 (CTLA-4), thereby suppressing effector T- and NK cells and reinforcing the immunosuppressive TME [99,100]. Stromal fibroblasts are activated by tumor necrosis factor alpha (TNF-α) and TGF-β derived from HRS cells and other infiltrating cells [101]. They synthesize collagen that forms the dense fibrous stroma characteristic of nodular sclerosis [101]. Collagen engagement of discoidin domain receptor (DDR)1 (and likely DDR2) on HRS cells increases resistance to apoptosis and reduces therapeutic sensitivity [102]. Below, we summarize the cellular and paracrine interactions in the TME (Fig. 1).

### 2.4 Role of CD30 in cHL

CD30 is a membrane receptor from the tumor necrosis factor receptor (TNFR) superfamily, encoded by the *TNFRSF8* gene [103,104]. It was first identified in 1982 by the group of Harald Stein using the monoclonal antibody Ki-1 on HRS cells from patients with Hodgkin lymphoma [105]. CD30 is a type I transmembrane glycoprotein of 595 amino acids (molecular weight 105–120 kDa) with an extracellular domain containing six characteristic cysteine-rich repeats, a single transmembrane segment, and a cytoplasmic signaling domain [106,107].

Under physiological conditions, CD30 expression is highly restricted, occurring in a small subpopulation of activated T- and B-lymphocytes [108,109]. By contrast, many lymphoid neoplasms display aberrantly high CD30 expression, with the highest levels in tumor HRS cells in cHL and in Anaplastic Large Cell Lymphoma (ALCL) [110,111]. Variable CD30 expression is also observed in other lymphoproliferative diseases, including some peripheral T-cell lymphomas (PTCL), cutaneous T-cell lymphomas (e.g., Mycosis Fungoides), and even certain B cell tumors such as subsets of diffuse large B cell lymphoma (DLBCL) [107,112,113]. CD30 can also be induced on lymphocytes during specific viral infections, including Human T-cell Leukemia Virus 1 (HTLV-1), HIV-1, and EBV [114,115]. This pattern supports the view that CD30 is a marker of lymphoid cell activation.

CD30 serves as the receptor for its cognate ligand CD30L (also known as CD153), a membrane protein of the tumor necrosis factor family expressed mainly on activated immune cells (including T-lymphocyte subpopulations, histiocytes, and granulocytes) [116]. Binding of trimeric CD30L induces receptor trimerization and recruitment of adaptor proteins from the TRAF (TNF receptor-associated factors) family to the cytoplasmic domain of CD30 [117]. This, in turn, activates intracellular signaling cascades—primarily NF-κB and MAPK, including Extracellular Signal-Regulated Kinase 1/2 (ERK1/2)—that drive programs of survival, proliferation, and cytokine secretion [118–120]. Accordingly, CD30 stimulation exerts a pronounced pro-survival, anti-apoptotic effect in CD30-expressing cells.

Malignant HRS cells within the cHL microenvironment overexpress CD30, which can lead to constitutive, ligand-independent activation of downstream pathways [121]. CD30 overexpression has been shown to induce spontaneous activation of NF-κB in HRS cells, supporting uncontrolled growth and resistance to apoptosis [28,122]. Cells in the microenvironment (e.g., activated T-lymphocytes and macrophages) also express CD30L and engage CD30 on HRS cells [123]. Experimental studies demonstrate that CD30L binding enhances activation of Hodgkin tumor cells and stimulates cytokine secretion, whereas in ALCL cells the same signal can induce apoptosis [120,124]. Taken together, these observations indicate that CD30-CD30L interactions within the HL microenvironment contribute substantially to pathogenesis by promoting HRS-cell proliferation and remodeling the immune milieu.

## 3 Anti-CD30 Targeted Immunotherapy in cHL

Despite high overall cure rates in cHL, up to 30% of patients develop R/R disease, particularly after failure of high-dose chemotherapy and ASCT [62,125,126]. This unmet need has driven the search for targeted therapies capable of achieving durable disease control. CD30 is a clinically validated target in cHL: it is a defining marker of HRS cells, exhibits high and relatively uniform expression in most cases, and shows limited distribution in normal tissues, features that favor selective drug delivery [127,128]. In physiological conditions, CD30 is transiently expressed on subsets of activated T- and B-lymphocytes within secondary lymphoid tissues (e.g., paracortical/interfollicular zones of lymph nodes and tonsil) and in the thymic medulla, while resting lymphocytes and most non-hematopoietic tissues show minimal to absent expression [129,130]. Clinical experience has already validated this approach through BV, an anti-CD30 monoclonal antibody conjugated to the antimitotic payload monomethyl auristatin E (MMAE) [9]. BV has shown substantial activity in R/R cHL and systemic ALCL, producing meaningful response rates even in heavily pre-treated cohorts [131]. This success underscores the therapeutic relevance of CD30 and has spurred the development of next-generation CD30-directed modalities, including CAR-T cells and antibodies, which aim to provide more effective and selective treatment options for CD30^+^ lymphomas.

In lymphoma clinical trials, response is assessed using criteria distinct from those for solid tumors (e.g., RECIST). In Hodgkin lymphoma, early studies frequently used the Cotswolds-modified Ann Arbor criteria for staging and response definitions [132]. Standardization began with IWG-1999 criteria (International Working Group, 1999), which used computed tomography (CT)-based bidimensional measurements with the sum of the products of perpendicular diameters (SPD) and the categories complete response (CR), partial response (PR), stable disease (SD), and progressive disease (PD), introducing complete response, unconfirmed (CRu) [133]. IWG-2007 (Revised Response Criteria for Malignant Lymphoma) incorporated fluorodeoxyglucose (FDG)-PET for FDG-avid disease, eliminated CRu, and allowed immunohistochemistry (IHC) and bone marrow flow cytometry (FCM); CT-based SPD remained acceptable when needed, thereby expanding the role of PET compared with IWG-1999 criteria [134]. In 2009, the Deauville five-point PET scale—a qualitative visual scale that compares lesion uptake with mediastinal and hepatic reference activity—standardized FDG-PET interpretation for interim and end-of-treatment assessment, replacing the *de facto* binary PET-positive/negative approach used after IWG-2007 criteria [135]. The Lugano 2014 consensus formalized PET/CT as the standard for FDG-avid lymphomas with use of the Deauville scale, retained CT-based SPD for non-FDG-avid disease or when PET is unavailable, and updated staging recommendations; its key advance over prior systems was embedding PET/CT and Deauville within the response criteria [136]. Finally, RECIL 2017 (Response Evaluation Criteria in Lymphoma) introduced a unidimensional approach using the sum of longest diameters (SLD) of up to three target lesions and added the category minor response (MR; a 10%–30% decrease in SLD), thereby differing from Lugano criteria by moving from SPD to SLD, reducing the number of target lesions, and adding a new response grade [137]. In this review, we explicitly report the response-assessment criteria for each trial (when available) to enable valid cross-trial comparisons.

Monoclonal antibodies (mAbs) targeting CD30 were the first modality to demonstrate the clinical feasibility of CD30-directed therapy in cHL [138]. As drugs, antibodies exert therapeutic effects through several mechanisms: direct induction of apoptosis, complement-dependent cytotoxicity (CDC), antibody-dependent cellular cytotoxicity (ADCC), and modulation of the TME [139,140]. Although early unconjugated anti-CD30 mAbs (e.g., SGN-30, MDX-060) showed only modest clinical activity in early-phase studies, they paved the way for more effective formats, including antibody-drug conjugates and engineered multivalent or bispecific molecules [141,142]. Moreover, mAbs provide the scaffold for contemporary bispecific and multivalent architectures that are under active development among antibody-based therapeutics.

### 3.1 Antibody-Drug Conjugate

BV (SGN-35), marketed as Adcetris, is the first antibody–drug conjugate (ADC) in clinical practice developed for the targeted therapy of CD30-positive lymphomas, including cHL and systemic ALCL [143]. In the early 2000s, Seattle Genetics, Inc. developed SGN-35, a human chimeric immunoglobulin G1 antibody directed against CD30 that is covalently conjugated to the potent microtubule-disrupting agent MMAE via a protease-cleavable Val-Cit peptide linker [144]. The free MMAE binds tubulin, disrupting microtubules, causing G2/M cell-cycle arrest and triggering intrinsic apoptosis (e.g., caspase-3 activation) in the tumor cell [145–147]. This cytolysis can induce endoplasmic-reticulum stress and an immunogenic cell death, releasing tumor antigens and danger signals that recruit DCs and T-cells into the lymphoma microenvironment [148]. Importantly, membrane-permeable MMAE can diffuse into neighboring cells, producing a “bystander” kill of antigen-negative tumor or stromal cells and partly overcoming the heterogeneity and immune-suppressive stroma of cHL [149,150]. The IgG Fc domain may also engage Fcγ receptors on NK cells or macrophages, mediating antibody-dependent cellular phagocytosis (ADCP)/ADCC and further enhancing antitumor immunity [151]. Peripheral neuropathy, which results from MMAE’s disruption of axonal microtubules in peripheral nerves, is the most frequent dose-limiting toxicity [152]. Hematologic toxicity, such as neutropenia and other cytopenias (e.g., from bone marrow suppression), is also common, and infusion-related reactions are observed in a minority of patients, typically mild though severe anaphylaxis may rarely occur [153]. Initial preclinical studies demonstrated sustained tumor regression in CD30^+^ lymphoma models, supporting advancement to clinical trials.

A key milestone was a Phase I clinical trial (NCT00430846), in which BV showed high activity in patients with refractory HL and ALCL with a manageable toxicity profile [154]. The subsequent Phase II trial (NCT00848926) confirmed efficacy in R/R cHL after ASCT, with an ORR of 76/101 (75.2%), comprising CR 35/101 (34.6%) and PR 41/101 (40.6%); SD occurred in 22/101 (21.8%) and PD in 3/101 (3.0%), per IWG-2007 criteria [155]. These results led to accelerated Food and Drug Administration (FDA) approval in August 2011 for R/R cHL and ALCL, with European Medicines Agency (EMA) approval for BV following in October 2012.

In subsequent years, the clinical use of BV has broadened: it has been evaluated as post-transplant consolidation (after ASCT) and in combination regimens with immuno-oncology agents, including PD-1 checkpoint inhibitors [156]. A pivotal Phase III trial, ECHELON-1 (NCT01712490), showed that combination of BV and adriamycin, vinblastine, dacarbazine (AVD) significantly improved PFS compared with standard ABVD in patients with stage III–IV cHL, supporting incorporation of BV into current treatment guidelines [157]. A comprehensive review of BV trials and combinations is beyond the scope of this article; recent manuscripts provide detailed summaries [158].

### 3.2 Unconjugated Antibodies

Unconjugated anti-CD30 monoclonal antibodies bind CD30 on tumor cells and either directly modulate signaling, inducing G1 cell-cycle arrest and apoptosis by attenuating NF-κB–dependent survival pathways, or act via Fc-mediated effector functions, predominantly ADCC (through NK cells/monocytes) and ADCP (through macrophages via FcγRIIIa/CD16a) [159,160]. Within the TME, NK cells and macrophages constitute the principal effectors whose abundance, activation state, and FcγRIIIa polymorphism shape response magnitude, whereas constitutively active NF-κB and immunosuppressive stromal cues correlate with reduced sensitivity [161].

#### 3.2.1 SGN-30 Monoclonal Antibody

The first anti-CD30 agent developed by Seattle Genetics, Inc. was SGN-30, a monoclonal antibody that demonstrated activity against CD30^+^ lymphomas prior to the development of BV [162]. In preclinical studies, SGN-30 showed high efficacy against L-540, KM-H2, HDLM-2, and L-428 cell lines *in vitro*, inducing G1 cell-cycle arrest and apoptosis. In SCID (C.B-17) mice bearing L540Cy xenografts, SGN-30 at 4 mg/kg yielded 100% survival through at least 120 days, even when treatment initiation was delayed, whereas in a subcutaneous HL model it produced marked, dose-dependent tumor-growth inhibition [159]. Fc-mediated activation of macrophages via ADCP was identified as a critical component of SGN-30 activity *in vivo* [163]. However, in two clinical studies—NCT00337194 (Phase II, SGN-30 plus chemotherapy) and NCT00051597 (Phase I/II, SGN-30 monotherapy)—in patients with R/R cHL, no ORR was observed; only SD was reported in 28.9% and 19.0% of participants, respectively, with antitumor responses defined by the Cotswolds criteria for HL [142,164]. Despite limited clinical efficacy, the favorable safety profile of SGN-30 helped lay the groundwork for the development of ADCs, notably SGN-35.

#### 3.2.2 MDX-060 Monoclonal Antibody

MDX-060, also known as iratumumab, is a fully human anti-CD30 IgG1κ monoclonal antibody (clone 5F11) developed by Medarex, Inc. that specifically recognizes epitopes within CD30 cluster A and exhibits high affinity for its target. *In vitro*, MDX-060 effectively mediated ADCC by activated human peripheral blood monocytes, producing dose-dependent lysis of L-540 HL cells; upon cross-linking, the antibody also inhibited tumor-cell growth by reducing cell viability. *In vivo*, MDX-060 demonstrated antitumor activity: in a subcutaneous L-540Cy xenograft model in SCID mice, treatment produced significant tumor-growth delay and complete regressions in 40% of animals. In a more aggressive disseminated HL model, intravenous MDX-060 achieved long-term survival (up to 200 days) in 60% of mice as monotherapy and up to 100% when combined with a cross-linking antibody [165].

Additional studies showed that combining MDX-060 with chemotherapeutic agents (gemcitabine, etoposide, doxorubicin) yielded supra-additive effects against HL cells. The L-540 cell line was the most responsive: adding MDX-060 to gemcitabine halved the Half Maximal Inhibitory Concentration (IC50) of gemcitabine (0.17 to 0.08 μg/mL), and produced an additional 30%–40% reduction in viability compared with monotherapy. The combinations also enhanced apoptosis, with the apoptotic response correlating positively with CD30 expression and inversely with NF-κB activity. Consistent with this, HL cell lines with high NF-κB (e.g., L-428, L-1236) were less sensitive to MDX-060 both as monotherapy and in combination, suggesting a role for this transcription factor in drug resistance [166].

The subsequent clinical trial NCT00059995 (Phase I/II) enrolled 72 patients with CD30^+^ lymphomas: 63 with R/R HL, 7 with ALCL, and 2 with CD30^+^ T-cell lymphoma. Iratumumab was administered as monotherapy at doses of 0.1–15 mg/kg once weekly for 4 weeks. Per IWG-1999 criteria, the ORR was 6/72 (8%): 2/72 (2.8%) patients with HL achieved PR and 4/72 (5.6%) achieved CR: two with HL and two with ALCL. SD occurred in 25/72 (34.7%) patients, and in five of these it persisted for more than 12 months. The safety profile was favorable: treatment-related adverse events (TRAE) of grade 3–4 occurred in 7% of patients (including dyspnea, anemia, and transaminase elevations), and the maximum tolerated dose (MTD) was not reached [141]. Thus, although MDX-060 was well tolerated, its limited efficacy as monotherapy curtailed further development. A similar Phase II study (NCT00284804) evaluating MDX-060 with gemcitabine has been completed, but results have not been published.

#### 3.2.3 MDX-1401 Monoclonal Antibody

MDX-1401, a nonfucosylated fully human anti-CD30 IgG1κ monoclonal antibody, demonstrated markedly improved functional activity compared with its parent, fucosylated analogue MDX-060. Removal of fucose residues increases MDX-1401 affinity for Fc gamma receptor IIIa (FcγRIIIa; also known as CD16a, expressed on NK cells and macrophages), encompassing both the high-affinity Val158 and low-affinity Phe158 alleles. In lymphoma cell models (including HL lines L-540, L-428, L-1236), MDX-1401 elicited higher ADCC, achieving maximal lysis of 87% vs. 63% with MDX-060, and retained activity in cells with low CD30 expression where MDX-060 was ineffective. In a systemic ALCL model in SCID mice, MDX-1401 at 1 mg/kg increased median survival to 57 days, compared with 32.5 days for MDX-060 and 29 days for controls. Thus, relative to its predecessor, MDX-1401 shows enhanced pharmacodynamic properties and greater antitumor efficacy even at low doses, across FcγRIIIa polymorphisms and a range of tumor CD30 expression levels [167].

The Phase I clinical trial NCT00634452 evaluated MDX-1401 in patients with R/R HL. No ORRs were observed; SD occurred in 8/12 patients (66.7%), including 2/12 (16.7%) showing ≥40% tumor reduction after two cycles (response-evaluation criteria not stated). PD occurred in 4/12 (33%), leading to early discontinuation. On-target pharmacodynamic activity was supported by a reduction in circulating CD30^+^ cells in 10/12 patients (83.3%) after one 4-week treatment cycle [168]. Full results have not yet been published.

#### 3.2.4 XmAb2513 Monoclonal Antibody

Xencor, Inc. developed a humanized anti-CD30 monoclonal antibody, XmAb2513, with an engineered Fc domain that increases affinity for FcγRIIIa by approximately 20-fold and thereby enhances ADCC. The original variable domain of chimeric antibody cAC10 was humanized using human string content optimization and combined with the modified Fc region. *In vitro*, on the CD30^+^ HL cell line L-540 using Peripheral Blood Mononuclear Cells (PBMC) effector cells (E:T = 25:1), XmAb2513 displayed about 3-fold greater potency than cAC10-IgG1 and 10-fold greater potency than 5F11, with a 4-fold higher maximal cytotoxic effect relative to cAC10-IgG1 [169].

The Phase I clinical trial NCT00606645 enrolled 23 patients with CD30^+^ cHL previously treated with ≥2 lines of therapy, including ASCT. XmAb2513 was administered at 0.3–12 mg/kg intravenous every 14 days. All 23 patients were assessed for safety; 19 were efficacy-evaluable. The ORR was 2/23 (8.7%), with both PRs at 9 and 12 mg/kg; SD occurred in 11/23 (47.8%) and PD in 10/23 (43.5%), per IWG-2007 criteria. Any decrease in tumor size from baseline was observed in 52% of patients. The MTD was not reached and no Dose-Limiting Toxicity (DLT) was identified. Adverse events occurred in all patients, 78.3% of which were treatment-related; the most common were fatigue, nausea, vomiting, and diarrhea (predominantly grade 1–2), and ≥grade 3 TRAEs occurred in 8.7% [170].

### 3.3 Bispecific Antibodies

Anti-CD30/CD3 bispecific engagers redirect cytotoxic T-lymphocytes to CD30^+^ targets, forming an immune synapse that activates ζ-chain ITAM–ZAP-70–LAT/SLP-76 signaling and downstream NFAT, NF-κB, and AP-1 programs to drive degranulation and pro-inflammatory cytokine release; these effects are further amplified by PD-1/SHP2 checkpoint blockade [171–173]. Innate-cell engagers such as anti-CD30/CD16 or anti-CD30/CD64 recruit NK cells or monocytes/macrophages through FcγRIIIa or FcγRI, triggering ITAM/Syk-dependent ADCC/ADCP and respiratory burst, amplifying Interferon gamma (IFN-γ)/TNF-α-rich chemokine milieus and enhancing effector infiltration while maintaining antigen-restricted activation [174,175].

#### 3.3.1 Anti-CD30/CD3

Bispecific anti-CD30/CD3 antibodies (biAbs), particularly the 8D10-OKT3 conjugate, demonstrated high-affinity, CD30-specific binding and effective T-cell activation *in vitro*. In multiple CD30^+^ models (including HL lines RPMI-6666 and L-428), T-cells armed with 8D10-biAb induced production of pro-inflammatory cytokines, IFN-γ, granulocyte macrophage colony-stimulating factor (GM-CSF), and TNF-α and mediated specific lysis of CD30^+^ tumor cells up to 80.3% at an E:T ratio of 32:1, while CD30^−^ cells were spared. In an NOD SCID Gamma (NSG) xenograft model (using CD30^+^ cells not derived from HL), infusion of armed T-cells significantly prolonged survival vs. controls. Notably, biAbs 8D10F10 showed strict specificity for CD30 when screened against more than 6000 human membrane proteins and exhibited non-overlapping epitope specificity relative to brentuximab, recognizing distinct epitope clusters on CD30 [176]. A Phase I clinical trial (NCT05544968; not yet recruiting) has been initiated to evaluate the safety and biological activity of anti-CD30/CD3 biAbs combined with autologous T-lymphocytes in patients with relapsed or refractory CD30^+^ neoplasms, including cHL.

In preclinical studies, DuoBody®-CD3xCD30 (GEN3017)—an Fc-silent bispecific IgG1 antibody generated using the DuoBody® platform and targeting CD3 and CD30—demonstrated potent antitumor activity against CD30^+^ hematological malignancies. When co-culturing healthy-donor T-cells with the L-428 cHL cell line for 72 h at an E:T ratio of 4:1, DuoBody-CD3xCD30 induced robust, dose-dependent T-cell cytotoxicity, reducing tumor-cell viability by >80% with a Half Maximal Effective Concentration (EC50) in the subnanomolar range. Cytotoxicity was accompanied by CD4^+^ and CD8^+^ T-cell activation and proliferation, and by production of proinflammatory cytokines. The effect required co-engagement of CD3 and CD30; control monospecific antibodies were ineffective [177].

The Phase I/IIa clinical trial NCT06018129 was designed to assess safety, tolerability, immunogenicity, and preliminary antitumor activity in patients with CD30^+^ lymphomas, including HL; however, it was discontinued in 2025 following a strategic portfolio reassessment by Genmab, Inc.

#### 3.3.2 Anti-CD30/CD64

H22×Ki-4 is a chemically linked biAb comprising the Fab^′^ fragment of the murine anti-CD30 IgG1 monoclonal antibody Ki-4 and the Fab^′^ fragment of the humanized anti-CD64 IgG1 monoclonal antibody H22. Expression of Fc gamma receptor I (FcγRI; CD64) is largely restricted to myeloid effector cells—monocytes, macrophages, and activated neutrophils—enabling selective recognition of antibody-opsonized tumor cells by cytotoxic effectors [178]. The H22 monoclonal antibody binds outside the ligand-binding site yet efficiently triggers FcγRI-mediated responses—including phagocytosis, respiratory burst, and ADCC—even in the presence of high concentrations of human serum IgG [179].

*In vitro*, using the L-540 (CD30^+^/CD64^−^) cell line and human monocytes, H22×Ki-4 mediated ADCC with tumor-cell lysis up to 51% at 0.1 μg/mL, comparable to the related A77×Ki-4 construct targeting CD89. In phagocytosis assays with monocyte-derived macrophages, H22×Ki-4 increased uptake of L-540 cells from a baseline of 45% to >75%, demonstrating robust enhancement of innate effector functions against tumor cells [180].

The Phase I trial enrolled 10 patients with CD30^+^ malignancies (8/10 R/R HL; 2/10 AITL), all treated with H22×Ki-4. Response was assessed per CT-based bidimensional criteria (SPD): CR in 1/10 (10%) and PR in 3/10 (30%); SD in 4/10 (40%), yielding an ORR of 4/10 (40%) and a clinical benefit rate of 8/10 (80%). TRAEs were grade 1–2 (fever, tachycardia, hypotension), and the MTD was not reached [181].

#### 3.3.3 Anti-CD30/CD16

HRS-3/A9 Monoclonal Antibody

The first preclinical studies of the bispecific murine IgG1 monoclonal antibody HRS-3/A9, which targets CD30 and CD16, demonstrated high specificity and efficacy both *in vitro* and *in vivo*, as reported by Hombach et al. In cell-based assays using CD30^+^ L-540, L-428, and HDLM-2 targets, co-culture with peripheral blood lymphocytes or NK-enriched populations in the presence of HRS-3/A9 induced specific lysis of L-540 cells of up to 35%, whereas the parental monospecific antibodies were inactive. No activity was observed against CD30^−^ HPB-ALL and L-735 cells. In a SCID mouse xenograft model with subcutaneous L-540Cy tumors, HRS-3/A9 administered together with human peripheral blood lymphocytes produced complete tumor regression in 10/10 animals by day 32, and long-term remission persisted in 40% of mice beyond 120 days. These data provided the first evidence that HRS-3/A9 can effectively redirect the cytotoxicity of unstimulated NK cells toward tumor targets [182].

In an early Phase I/II clinical trial of 15 patients with R/R cHL, response was assessed per CT-based bidimensional criteria (SPD): CR in 1/15 (6.7%), PR in 1/15 (6.7%), minor response in 3/15 (20.0%), SD in 2/15 (13.3%), and mixed response in 1/15 (6.7%); the remaining 7/15 (46.7%) had PD, for an ORR of 2/15 (13.3%) [183]. TRAEs occurred in 6/15 (40.0%), were limited to grade 1–2 (fever, lymph-node pain, rash, hypotension), and the MTD was not reached at the highest dose. An immune response to human anti-mouse antibodies (HAMA) developed in 6/13 evaluable patients without pre-existing HAMA (46.2%), comprising anti-idiotypic and anti-anti-idiotypic antibodies capable of binding CD30, consistent with induction of an idiotypic network after HRS-3/A9 administration [184].

AFM13 Monoclonal Antibody

The monoclonal antibody AFM13 (Acimtamig) represents the next step in the HRS-3/A9 lineage. It is a fully Fv-based tetravalent bispecific antibody (TandAb) with humanized anti-CD30 and fully human anti-CD16a domains. Unlike IgG1-containing antibodies, the TandAb lacks an Fc domain, eliminating nonspecific FcγR-mediated activation and ensuring strictly antigen-dependent recruitment of NK cells. In preclinical studies, AFM13 recruited NK cells and induced lysis of CD30^+^ tumor cells. Across CD30^+^ lines, including four HL models (L-428, L-1236, HDLM-2, L-540Cy), the TandAb exhibited EC50 values of 3–39 pM, on average 10–15-fold more potent than a bispecific diabody and an Fc-enhanced IgG. The TandAb also showed prolonged retention on NK cell surfaces and maintained cytotoxic activity after washout of free antibody. Its activity required CD30^+^ targets and did not trigger nonspecific activation or bystander-cell lysis [185].

In preclinical *in vitro* models, Zhao et al. showed that AFM13, in co-cultures with PBMCs or NK-enriched fractions, produced dose-dependent lysis of lymphoma cells up to 40%, whereas monotherapy with immunomodulatory antibodies achieved less than 25% lysis. Combining AFM13 with anti-PD-1 or anti-CD137 increased cytotoxicity to 70%, and concurrent use of both antibodies yielded the largest combined effect. In an *in vivo* PDX model of cHL (Rag2^−^/^−^IL2Rγ^−^/^−^ mice bearing human CD30^+^ tumors with autologous PBMCs), AFM13 significantly inhibited tumor growth; by day 58, the anti–PD-1 combination induced tumor regression in 3/4 cases. Treatment also increased CD8^+^ T-cell infiltration approximately 16-fold and NK cells activation up to 32-fold vs. control, with elevated IFN-γ, TNF-α, and IL-2 production [186].

In the Phase I trial NCT01221571, AFM13 monotherapy was administered to 28 previously treated patients with R/R cHL (78.6% had received high-dose chemotherapy with autologous transplantation; 28.6% had received BV). Response was assessed per IWG-2007 criteria. Among 26 evaluable patients, ORR was 3/26 (11.5%; PR only), SD 13/26 (50%), and PD 10/26 (38.5%), yielding a disease control rate of 61.5%. Among BV-refractory patients, SD was achieved in 85.7%. Pharmacodynamic analyses showed significant NK-cell activation and a decrease in soluble CD30 in peripheral blood. The safety profile was favorable: TRAEs ≥grade 3 occurred in 28.6% of patients, with one DLT (hemolytic anemia) and no treatment-related deaths [187].

In the Phase Ib trial NCT02665650, AFM13 plus pembrolizumab was administered to 30 patients with R/R cHL. Response was assessed per Lugano criteria. All 30 were evaluable: investigator-assessed ORR was 25/30 (83%), comprising complete metabolic response (CMR) 11/30 (37%) and partial metabolic response (PMR) 14/30 (47%); no metabolic response (NMR) occurred in 2/30 (7%) and PD in 3/30 (10%). At the highest dose (7 mg/kg AFM13 plus pembrolizumab 200 mg every 3 weeks; cohort 3 + extension; n = 24), ORR was 21/24 (88%) by both investigator and independent assessments; by investigator review, CMR was 10/24 (42%), PMR 11/24 (46%), NMR 2/24 (8%), and PD 1/24 (4%), while by independent review CMR was 11/24 (46%), PMR 10/24 (42%), and PD 3/24 (13%). The most common TRAEs were infusion reactions, rash, and nausea; ≥grade 3 TRAEs occurred in 23%. The combination showed a manageable safety profile and high activity, including in BV-refractory patients [188].

AFM13 with Adoptive NK Cells

A rational strategy to further enhance activity is combination with adoptive NK cell infusions. Recent preclinical studies evaluated AFM13 with AlloNK (AB-101), an allogeneic cryopreserved NK cell product enriched for the CD16a 158V/V variant. *In vitro*, against the CD30^+^ Karpas-299 line, the combination induced specific tumor-cell lysis up to 80% and increased NK cell degranulation marker expression (CD107a) and IFN-γ production by more than twofold. In NOG-hIL-15 mice bearing Karpas-299/Luc xenografts (disseminated lymphoma model), the combination significantly suppressed tumor growth; AFM13 bound about 90% of CD56^+^CD16^+^ AB-101 NK cells without fratricide and with preserved viability [189].

In the Phase II clinical trial NCT05883449 (LuminICE-203), AFM13 combined with cryopreserved allogeneic NK cells, AlloNK (AB-101), was administered to 10 patients with R/R cHL previously treated with chemotherapy, PD-1 inhibitors, and BV; 50% had undergone ASCT. Response was assessed per Lugano criteria. The ORR was 9/10 (90%), comprising CR in 6/10 (60%) and PR in 3/10 (30%); PD occurred in 1/10 (10%). The safety profile was acceptable: TRAEs occurred in all patients, most commonly infusion reactions (60%) and cytokine release syndrome (CRS, 30%), predominantly mild to moderate. No cases of Immune Effector Cell-Associated Neurotoxicity Syndrome (ICANS), Graft-vs.-Host Disease (GvHD), or treatment discontinuation due to adverse events were observed [190].

*In vitro*, AFM13 combined with IL-12/15/18–activated NK cells induced multifunctional activation, enhancing IFN-γ and TNF-α production and increasing CD107a degranulation. Pre-activated, expanded NK cells from umbilical cord blood showed a 1.8-fold increase in specific lysis of Karpas-299 (CD30^+^) cells vs. control. AFM13–NK CAR-like complexes retained cytotoxicity for >72 h after incubation and demonstrated durable activity *in vivo*: in NSG mice bearing a Karpas-299 xenografts, treatment produced a 3.4-fold reduction in tumor bioluminescence signal and improved survival without signs of toxicity. The AFM13–NK complex mediated targeted cytotoxicity without genetic engineering, effectively mimicking CAR-like activity [191].

In the subsequent Phase I/II trial NCT04074746, combination therapy with AFM13 and pre-activated, expanded umbilical cord blood-derived NK cells were evaluated in 42 patients with refractory CD30^+^ lymphomas (37 with cHL; 5 with CD30^+^ non-Hodgkin lymphoma) who had received a median of seven prior lines and were refractory to BV. Response-evaluation criteria not stated. At the recommended phase 2 dose of 1 × 10^8^ NK cells/kg (n = 36), ORR was 34/36 (94.4%), comprising CR 26/36 (72.2%) and PR 8/36 (22.2%); SD 2/36 (5.6%) and PD 0/36 (0%). In the overall cohort (n = 42), ORR was 39/42 (92.8%), including CR 28/42 (66.7%). Nine patients had responses consolidated with stem-cell transplantation (5 allogeneic, 4 autologous). The safety profile was favorable: no CRS, ICANS, or GvHD were observed. This combination demonstrated marked clinical activity and excellent tolerability in heavily pretreated patients, including those who subsequently underwent consolidative transplantation [192].

#### 3.3.4 Anti-CD30/CD137

In preclinical studies, a bispecific recombinant IgG1 antibody in the CrossMab format targeting CD30 and CD137, co-expressed on HRS cells, was developed. CD137 expression on HRS cells has been reported in 57%–86% of HL cases [193]. The antibody was tested on HL cell lines (KM-H2, HDLM-2, L-428, L-1236), in which CD30^+^CD137^+^ cells comprised 84%–87%. The selected antibody clone 2 induced NK-mediated ADCC, producing a fourfold increase in lysis of CD30^+^CD137^+^ cells vs. isotype control, whereas lysis of single-positive populations did not exceed baseline levels. In KM-H2 cells, the same clone directly induced apoptosis, increasing early apoptosis from 5.6% to 21.9% and late apoptosis from 3.9% to 8.7%. The antibody also underwent efficient internalization in double-positive cells, supporting its use as a payload-delivery platform analogous to BV [194].

In contrast to the previously described T- and NK-cell engagers, the CD30/CD137 mAb employs a dual tumor-antigen targeting paradigm: co-binding CD30 and CD137 on the same HRS cell increases avidity and selectivity, enhances Fc-dependent NK-mediated ADCC, and supports efficient internalization for potential payload delivery, rather than providing costimulatory activation of effector cells. This mechanistic distinction underlies the preferential cytotoxicity against CD30^+^CD137^+^ subpopulations and positions CD30/CD137 constructs primarily as tumor-binding and delivery platforms rather than classic immune-cell redirectors. Immune-cell mAb engagers form a direct effector–target synapse, enabling more immediate cytotoxicity and yielding encouraging early clinical signals; however, no head-to-head comparisons with tumor-binding, non-engager bispecifics (e.g., CD30×CD137) have been reported.

### 3.4 Radioimmunoconjugates

CD30-directed radioimmunoconjugates (RICs) deliver ionizing radiation to cHL cells, inducing DNA double-strand breaks and activating the DNA damage response involving ATM, ATR, CHK1/2 and p53 signal pathway; this cascade promotes apoptosis and immunogenic cell death with exposure of DAMPs, activation of cGAS–STING and interferon signaling, and upregulation of PD-L1 [195–197].

In a preclinical study, anti-CD30 RICs were evaluated in SCID mice bearing human cHL xenografts. The murine IgG1 antibody Ki-4 and the humanized IgG3 antibody 5F11 were radiolabeled either directly with Iodine-131 using the chloramine-T method or with Indium-111 via p-isothiocyanatobenzyl-DOTA chelation; an iodinated Ki-4 diabody was also tested. *In vitro*, all constructs retained high CD30 binding affinity with dissociation constants in the range of approximately 20 to 220 nanomolar, as measured on CD30^+^ L-540 cells. *In vivo*, tumor uptake at 24 h post injection spanned approximately 2.6% to 12.3% injected dose per gram, with the highest value observed for iodine-131–Ki-4; however, blood activity remained high and tumor-to-blood ratios were below one at all measured time points. Despite strong CD30 binding, biodistribution was suboptimal, which indicates that *in vivo* kinetics require optimization through antibody format engineering and pharmacokinetic modulation. Subsequent radioimmunotherapy programs have explored strategies such as pre-dosing with unlabeled antibody to mitigate circulating antigen sinks and improve tumor-to-background contrast [198].

In a Phase I feasibility study, radioimmunotherapy with an iodine-131–labeled murine anti-CD30 monoclonal antibody (¹³¹I–Ki-4) was administered to 22 patients with R/R CD30^+^ HL; patients were heavily pretreated—16/22 had prior high-dose chemotherapy with ASCT and 17/22 had prior radiotherapy. At dosing, patients received a 5 mg unlabeled Ki-4 preinfusion, a day 1 dosimetric infusion of 250–300 MBq ¹³¹I–Ki-4, then a therapeutic infusion on day 8 (median 2332 MBq; range 743–3823 MBq), yielding total-body doses of 0.035–0.989 Gy; preinfusion bound circulating soluble CD30 in about 91% of patients. Across the full cohort (n = 22), ORR was 6/22 (27.3%), comprising CR 1/22 (4.5%) and PR 5/22 (22.7%); additional outcomes included MR 3/22 (13.6%), SD 1/22 (4.5%), and PD 12/22 (54.5%). Median response duration was about 4 months (CR 5 months; PR up to 6 months) with antitumor responses defined by the Cotswolds criteria for HL. Infusions were generally well tolerated (fatigue grade 1 in 20/22; mild nausea in 5/22; isolated grade 1 pain/gastritis; one allergic reaction; one skin ulcer; one hemorrhagic colitis of uncertain relatedness). Hematologic toxicity was dose-limiting: among 21 assessable patients, grade 4 events occurred in 7/21 (33.3%), thrombocytopenia in 7, neutropenia in 3, anemia in 1. Human anti-mouse antibodies developed in 4/22 by day 7 and were associated with shortened radiotracer residence when treatment was delayed. Overall, ¹³¹I–Ki-4 showed modest antitumor activity with substantial myelosuppression risk in heavily pretreated HL [199].

Following the ¹³¹I–Ki-4 experience, no other completed clinical trials of CD30-targeted RIT in cHL have been published. A Phase I trial of ^90^Y-HeFi-1 (with ¹¹¹In-HeFi-1 for imaging/dosimetry) initiated in the late 2000s has not reported results, despite the theoretical advantage of radiometals owing to intracellular retention after antibody internalization; no active NCT registrations for CD30-RIT are identifiable, and reviews note that the approach has not advanced clinically since ¹³¹I–Ki-4 [200]. Other RIT strategies have been tested in related contexts (e.g., anti-CD25 ^90^Y-daclizumab), but for CD30 in cHL no programs remain; by 2025 the field has been effectively displaced by the success of antibody-drug conjugates and immune checkpoint inhibitors [201,202].

### 3.5 Alternative Approach: Oncolytic Viruses

Oncolytic virotherapy uses replication-competent viruses engineered to preferentially infect and lyse CD30^+^ HRS cells. Once inside the tumor cell, the virus replicates until inducing lytic cell death. Viral replication releases pathogen-associated molecular patterns (PAMPs) and tumor damage signals that activate intracellular innate sensors (e.g., RIG-I, MDA5, TLR3) in infected cells and APCs [203]. This triggers robust type I interferon and pro-inflammatory cytokine cascades, promoting dendritic-cell maturation and NK-cell activation. The dying tumor cells expose viral and tumor antigens, leading to immunogenic cell death—a source of tumor-associated antigens that prime adaptive immunity [204]. In this way, oncolysis converts the “cold” HL microenvironment into a “hot” one: it increases antigen presentation and effector T-/NK-cells infiltration, induces pro-inflammatory chemokines, and can normalize tumor vasculature and matrix [205].

In a preclinical study, Hanauer et al. demonstrated the efficacy of retargeted oncolytic viruses directed to CD30^+^ cHL cells. Using an optimized scFv derived from HRS-3 antibody (HRS3opt2#2), the team engineered CD30-targeted measles virus (MV-CD30) by fusing the construct to a defective H protein, and vesicular stomatitis virus (VSV-CD30) by complete replacement of the glycoprotein G gene. *In vitro*, cHL cell lines, including KM-H2, L-428, and L-1236, displayed variable CD30 expression but were susceptible to infection by both viruses; VSV-CD30 was most potent, reducing KM-H2 viability by 52% at 48 h. In NSG mice bearing KM-H2 xenografts, systemic or intratumoral VSV-CD30 infusion significantly slowed tumor growth and prolonged survival: 100% of treated mice exhibited complete suppression of tumor growth for several weeks, whereas control group reached the endpoint over the same interval. In a disseminated-tumor model, 5/8 animals showed decreased bioluminescence, confirming activity against systemic disease [206].

### 3.6 CAR-T Cell Therapy

CAR-T cells targeting CD30 are among the most promising cellular therapies for cHL. The advent of CAR-T technology enabled directing T-cell cytotoxicity against CD30^+^ targets independent of MHC, thereby overcoming immune-evasion mechanisms [207]. Upon binding CD30, CAR activate TCR-like intracellular signaling: phosphorylation of CD3ζ ITAMs recruits ZAP-70, driving NFAT, NF-κB and AP-1 and inducing cytotoxicity and cytokine production [208]. Second-generation CARs add a CD28 or 4-1BB co-stimulatory domain, which provides survival and proliferative signals (via PI3K/Akt and TRAF2/NF-κB pathways) [209]. In clinical trials, 28ζ vs. 28BBζ CARs had markedly higher IL-2 and IFN-γ release and longer T-cell persistence [210]. CAR-T–secreted IFN-γ and TNF-α can upregulate MHC and chemokines in the tumor, recruiting macrophages and cross-priming DCs [211].

Iterative design, from first-generation receptors to second- and third-generation systems with costimulatory domains, has aimed to improve persistence and antitumor activity across multiple preclinical and clinical studies. Detailed discussions of the functional effects of distinct costimulatory domains (e.g., CD28 and 4-1BB) across CAR-T generations, as well as safety considerations, including CRS and ICANS, are available in recent comprehensive reviews [212,213].

#### 3.6.1 First-Generation CAR-T Preclinical Studies

The development of first-generation CARs logically extended earlier efforts to redirect T-cell responses to defined tumor antigens without MHC restriction [214,215]. These constructs comprised an extracellular antigen-binding domain based on a single-chain variable fragment (scFv), a hinge region, a transmembrane domain, and an intracellular CD3ζ signaling module that initiated a T-cell receptor (TCR)-like cascade [216,217]. This architecture provided target recognition via the scFv, flexibility via the hinge, membrane anchoring via the transmembrane domain, and signal transduction via CD3ζ; however, the absence of costimulatory signals limited *in vivo* persistence and functional activity [218,219].

Savoldo et al. first developed an immunotherapy strategy for HL based on transducing EBV-specific cytotoxic T-lymphocytes (EBV-CTLs) with a CD30-directed CAR. The resulting CD30CAR^+^ EBV-CTLs retained native specificity for EBV antigens (65% ± 14% lysis of autologous lymphoblastoid cell lines, LCLs) and acquired the ability to kill EBV^−^/CD30^+^ tumor cells (up to 58% ± 21% specific lysis of HDLM-2, L-428, and KM-H2 at E:T = 20:1). In a SCID mouse xenograft model, CD30.CAR^+^ EBV-CTLs induced sustained regression of L-428 tumors, achieving complete eradication in 30% of animals; prolonged T-cell expansion occurred only with EBV-specific stimulation. Notably, CAR^+^ cells remained effective in the presence of soluble CD30 and did not impair endogenous T-cell effector functions, as evidenced by preserved recall responses to cytomegalovirus and adenovirus. These data suggest that dual specificity affords complementary tumor control and may reduce immune escape, supporting CD30.CAR^+^ EBV-CTLs as a promising platform for treating R/R cHL [220].

#### 3.6.2 Second Generation CAR-T Preclinical Studies

The second generation of CARs was developed to overcome first-generation limitations, most notably limited persistence and suboptimal *in vivo* function. These constructs add an intracellular costimulatory domain (most commonly CD28 or 4-1BB) to the CD3ζ signaling module, providing additional activation through the phosphoinositide 3-kinase (PI3K)/AKT serine-threonine kinase (AKT) and NF-κB pathways, thereby enhancing proliferation, survival, and cytokine production [221–223]. The antigen-binding domain, hinge, and transmembrane regions retain roles analogous to first-generation designs, while the costimulatory module is encoded within the same transgene as CD3ζ, ensuring co-delivery of both signals upon target engagement. These refinements produced marked gains in expansion, persistence, and antitumor activity in preclinical models and now constitute the predominant architecture for clinical CAR-T products [224,225].

Di Stasi et al. first developed second-generation CAR-T cells co-expressing a CD30-directed CAR and CCR4 to enhance homing to tumors in cHL. The rationale was the high expression of CCL17 and CCL22 by tumor cells (HDLM-2, L-428, L-1236), which physiologically attract CCR4^+^ Tregs and Th2 cells, whereas CD8^+^ effector T-cells lack CCR4 and infiltrate poorly. Introducing CCR4 into anti-CD30 CAR-T cells markedly improved chemotaxis *in vitro* (migration toward HDLM-2 increased from 4.7% to 40.0%) without altering phenotype or imparting suppressive properties. In a SCID mouse model with subcutaneous implantation of CCL17-secreting Karpas-299 cells, intratumoral accumulation of CCR4^+^ anti-CD30 CAR-T cells increased (4.4 × 10^5^ vs. 5 × 10^4^ p/s/cm^2^/sr). Moreover, in NOG/SCID/γc^−^/^−^ mice bearing HDLM-2 tumors, CCR4^+^ anti-CD30 CAR-T cells controlled tumor growth, with 57% of mice showing tumor regression 30 days after infusion [226].

Alvarez-Fernandez et al. developed stem-like memory T-cells (T_SCM_) expressing an optimized anti-CD30 CAR that demonstrated significant antitumor activity in preclinical cHL models. The CAR targeted a membrane-proximal, non-cleavable CD30 epitope, conferring resistance to inhibition by soluble CD30. T_SCM_ were generated from naive human CD4^+^/CD8^+^ T-cells activated via CD3/CD28 and cultured with IL-7, IL-15, and IL-21. *In vitro*, anti-CD30 CAR-T_SCM_ mediated antigen-specific cytotoxicity against cHL cell lines L-540 and L-428 (lysis 89.9% and 71.1%, respectively, at E:T = 5:1) and maintained activity in the presence of 20 μg soluble CD30. In NSG mice bearing systemic (L-428, intravenous) or subcutaneous (L-540) lymphoma models, infusion of 1 × 10^7^ anti-CD30 CAR-T_SCM_ achieved complete tumor eradication in 100% of animals (n = 4), with persistence in bone marrow and lymph nodes (up to 75.5% CAR^+^ T-cells) and durable immunity (100% survival after rechallenge on day 79). Products enriched for T_SCM_ (>70%) outperformed those with lower T_SCM_ content, yielding higher tumor infiltration (78.8% vs. 53.8% CAR^+^ T-cells) and greater circulating CAR-T frequencies (740 vs. 138 CAR^+^ cells/μL) [227].

Guo et al. developed a CD30-directed CAR based on a humanized scFv (hHRS3-CAR) and demonstrated high efficacy in preclinical cHL models. In cell-based assays, hHRS3 CAR-T cells efficiently killed CD30^+^ tumor lines (L-428, L-540) but not CD30^−^ control lines (Raji, U937), achieving ≥80% specific lysis at an E:T = 5:1 ratio. In immunodeficient NCG mice injected with 2 × 10^6^ L42-EGFP-luci cells, a single infusion of 1 × 10^7^ hHRS3 CAR-T cells produced a substantial reduction in tumor bioluminescence and extended median survival to 63 days vs. 40 days in controls. hHRS3 CAR-T cells also displayed an increased central memory (CCR7^+^CD45RO^+^) fraction *in vitro*, suggesting improved persistence *in vivo*. Overall, hHRS3 CAR-T cells showed cytotoxicity comparable to the original murine construct, with lower immunogenicity and a more favorable memory phenotype, supporting their potential for clinical application [228].

Beyond CD30, CAR-T cells can be directed to additional HRS-associated surface markers. Ruella et al. developed CD123-specific CAR-T cells (CART123) targeting the IL-3 receptor alpha chain. CD123 was detected on HRS cells and on TAMs in the TME, as shown by IHC and expression analyses in HL lines including HDLM-2, KM-H2, SUP-HD1, and L-428. *In vitro*, CART123 killed all four cHL lines, induced IFN-γ, TNF-α, and GM-CSF, and exhibited sustained proliferation. In immunodeficient NSG mice, administration of CART123 42 days after intravenous HDLM-2 engraftment produced complete tumor eradication within 14 days and 100% recurrence-free survival for 6 months, vs. a median survival of 128 days in controls. Upon rechallenge 250 days later, durable CART123 immune memory was confirmed. Notably, in the presence of immunosuppressive TAMs, CART123 retained function and eliminated CD123^+^ TAMs, whereas anti-CD19 CAR-T cells became suppressed under the same conditions [229].

Early efforts to make T-cell therapies resistant to tumor immunosuppression encompassed not only CAR designs incorporating costimulatory domains but also CAR-independent genetic platforms engineered to engage alternative signaling cascades [230]. Although these strategies are not formally categorized within CAR generations they laid the conceptual groundwork for subsequent incorporation of modules that sustain activation and augment the effector functions of engineered T-cells [230,231].

As an early example, Foster et al. enhanced anti-tumor responses by rendering T-cells resistant to tumor-derived TGF-β through transduction of EBV-CTLs with a dominant-negative TGF-β type II receptor (DNRII). Preclinical studies used lymphoma lines (including EBV^−^ HL-HDLM-2, L-1236, and EBV^+^-LCLs) and a SCID mouse model bearing EBV^+^ LCLs xenografts. In the presence of TGF-β1, proliferation of unmodified EBV-CTLs decreased by 80%, whereas DNRII-EBV-CTLs decreased by only 17%; similarly, HDLM-2 supernatant reduced proliferation by 53% in conventional EBV-CTLs vs. 12% in modified cells. In cytotoxic assays against LCL target, lysis at E:T = 20:1 ratio fell from 63% to 37% with TGF-β for unmodified EBV-CTLs, while DNRII-EBV-CTL activity was maintained or increased (61% to 73%). In SCID mice, DNRII-CTLs produced a sustained reduction in tumor bioluminescence and maintained antitumor activity for up to 28 days, whereas control T-cells were ineffective [232].

#### 3.6.3 Third Generation CAR-T Preclinical Studies

The third generation of CARs sought to further enhance T-cell proliferation, persistence, and antitumor activity by integrating two costimulatory domains, typically CD28 with 4-1BB or CD28 with OX40 into the construct [233,234]. This architecture retains the standard elements (scFv, hinge, transmembrane domain, CD3ζ) but enables concurrent activation of multiple signaling cascades, including PI3K/AKT, NF-κB, and MAPK, thereby prolonging activation, increasing cytokine production, and augmenting effector function *in vivo* [235,236]. Preclinical models indicate that such designs can outperform second-generation CARs in durability of response and reduced susceptibility to T-cell depletion, but their clinical advantage over prior generations remains under active investigation [237].

Zhang et al. developed a third-generation CD30-targeted CAR-T cell construct (CD30-28BBz) comprising a CD30-specific scFv (based on the patented sequence CN106589139B), a leader sequence with CD8 hinge, a CD8 transmembrane region, and intracellular CD28, 4-1BB (CD137), and CD3ζ signaling domains. Compared with a second-generation construct (CD30-28z), CD30-28BBz was designed for improved activity in R/R CD30^+^ lymphomas. *In vitro*, these CAR-T cells mediated selective cytolysis of CD30^+^ L-428 and L-540 tumor lines (≥50% lysis at E:T = 25:1 ratio) with no activity against CD30^−^ control lines (Raji, Jurkat) and absence of non-specific binding. In a subcutaneous xenograft model in NPG mice implanted with L-428, CD30-28BBz therapy significantly suppressed tumor growth: mean tumor volume was 4.6-fold lower than control at day 30, and overall survival was 50% at day 120. CD30-28BBz CAR-T cells exhibited prolonged persistence in blood and tumor relative to CD30-28z and showed preferential tumor infiltration (2.3-fold higher than in other tissues). Immunophenotyping revealed lower frequencies of PD-1^+^ and T-cell immunoglobulin and mucin-domain containing-3 (TIM-3)^+^ cells in the third-generation product, alongside a 37.5% higher level of IFN-γ vs. the second generation. Tumorigenicity and integration analyses showed no evidence of transformation and indicated preferential vector integration outside oncogenic regions [238].

#### 3.6.4 First-Generation CAR-T Clinical Studies

The earliest clinical trials of first-generation CARs in the late 1990s–early 2000s enrolled patients with melanoma, renal cell carcinoma, and neuroblastoma, but did not include anti-CD30 approaches for cHL [233]. These constructs contained only an antigen-binding domain and an intracellular CD3ζ module, enabling target recognition but yielding limited *in vivo* persistence and low efficacy [239]. Most studies reported only transient clinical responses, often without meaningful tumor reduction; in some cases, rapid T-cell attrition and weak cytokine production were noted [240].

#### 3.6.5 Second Generation CAR-T Clinical Studies

The insufficient efficacy of first-generation CARs, attributable to absent costimulation, motivated second-generation designs in which a costimulatory domain (e.g., CD28 or 4-1BB) was added to the CD3ζ module. Clinical trials of these constructs showed markedly improved persistence of modified T-cells, higher IL-2 and IFN-γ production, and objective responses, including complete remissions, in some patients with relapsed B cell malignancies [241–243]. These results established the central role of costimulatory signaling in enhancing the therapeutic activity of CAR-T cells and laid the groundwork for further platform refinements.

Early Second-Generation CD30 CAR-T Clinical Trials

The Phase I trial NCT03049449 evaluated a CD30-directed CAR-T cell product comprising a fully human anti-CD30 scFv (5F11), a CD28 hinge-transmembrane-costimulatory module, and a CD3ζ signaling domain in 21 patients with CD30^+^ lymphomas (20 with cHL; 1 with ALCL). After cyclophosphamide–fludarabine lymphodepletion, patients received anti-CD30 CAR-T cells at 0.3–9 × 10^6^ cells/kg. Response was assessed per IWG-2007 criteria: ORR 9/21 (43%), including CR 1/21 (4.8%) and PR 8/21 (38%); SD occurred in 11/21 (52%) and PD in 1/21 (4.8%). TRAEs occurred in all patients, including CRS in 11/21 (52%; grade 3 in 1/21), rash in 9/21 (43%; grade 3 in 3/21), neurologic toxicity in 5/21 (24%; all grade 2), and prolonged cytopenias in 5/21 (24%). Severe infections occurred in 2/21 (9.5%) in the context of prolonged cytopenias. The study was terminated early owing to toxicity and limited clinical efficacy [244].

The Phase I trial NCT04653649 evaluated the safety and preliminary efficacy of an autologous anti-CD30 CAR-T cell product generated by lentiviral transduction of T-cells with a second-generation, 4-1BB-costimulated CAR whose scFv targets a membrane-proximal, non-cleavable CD30 epitope, in 11 patients with R/R cHL (n = 9) or CD30^+^ T-cell non-Hodgkin lymphoma (n = 2). Of these, 10 received anti-CD30 CAR-T cells across three dose levels; the infused products were memory-enriched, with predominant CAR^+^ central memory T-cells (T_CM_) and T_SCM_ populations. Among infused patients, ORR was 100%: CR 5/10 (50%) and PR 5/10 (50%); no SD or PD was observed. Response was assessed per RECIL 2017 using FDG-PET/CT. TRAEs occurred in all patients: CRS in 6/10 (all grade 1), rash in 4/10, severe infections in 2/10, and prolonged cytopenias in 2/10; no neurotoxicity or DLT was reported. Anti-CD30 CAR-T cells were detectable in peripheral blood in all patients at 4 months and in all evaluable patients at 9 months [245].

The Phase I trial NCT02259556 evaluated the tolerability and activity of an autologous T-cell product expressing a CD30-directed CAR comprising an anti-CD30 scFv (accession AJ878606.1), a CD8α hinge, a transmembrane region, a 4-1BB (CD137) costimulatory domain, and a CD3ζ signaling domain. The study enrolled 18 patients (17 with cHL; 1 with ALCL), most with high disease burden. After lymphodepleting chemotherapy with fludarabine, patients received anti-CD30 CAR-T cells at a median dose of 1.56 × 10^7^ CAR^+^ T-cells/kg. Response was assessed per IWG-2007 criteria. ORR was 7/18 (39%; all PR); SD 6/18 (33%); PD 5/18 (28%); CR was not observed. TRAEs of grade ≥3 occurred in 2/18 (11%); the most common events were cytopenias, grade 1–2 fever, nausea, vomiting, and rash [246].

The Phase I trial NCT01316146 evaluated autologous anti-CD30 CAR-T cells comprising an scFv derived from the murine anti-CD30 antibody HRS-3, an IgG1 CH2-CH3 spacer, a transmembrane region, a CD28 costimulatory domain, and a CD3ζ signaling domain. Although the study is listed as withdrawn on ClinicalTrials.gov (no official site opening at the University of North Carolina), 9 patients with CD30^+^ lymphomas were treated on protocol: 6 with R/R cHL, 2 with ALCL, and 1 with composite DLBCL/HL. Anti-CD30 CAR-T cells were infused without lymphodepletion at three dose levels (0.2 × 10^8^ to 2 × 10^8^ cells/m²). Response was assessed per IWG-2007 criteria. ORR was 3/9 (33.3%; all CR); SD 3/9 (33.3%); PD 3/9 (33.3%). TRAEs were minimal: no CRS, no changes in B- or T-cell counts or virus-specific immune activity, and no subsequent infections were reported [247].

Lymphodepletion Strategies in CD30 CAR-T Therapy

The parallel Phase I/II trials (NCT02690545 and NCT02917083) enrolled 41 adults with R/R cHL. Participants received anti-CD30 CAR-T cells after lymphodepletion with fludarabine-containing regimens (n = 32) or bendamustine alone (n = 8). Response was assessed per Lugano criteria. Among patients with active disease who received fludarabine-containing lymphodepletion, the ORR was 72% (CR 59%, PR 13%), with SD 9% and PD 19%. In the bendamustine-only group, no ORRs were observed. Grade ≥3 TRAEs included lymphopenia, neutropenia, thrombocytopenia, and anemia; CRS occurred in 24% of patients, all grade 1 [248]. Thus, anti-CD30 CAR-T cell infusion after fludarabine-containing lymphodepletion showed high efficacy with a favorable safety profile in heavily pretreated cHL. In correlative analyses, baseline metabolic tumor volume (MTV) was the principal prognostic factor: patients with MTV >60 mL had worse 1-year PFS than those with MTV ≤60 mL (14% vs. 58%). Neither CAR-T cell expansion in peripheral blood nor PD-1 expression on CD3^+^ lymphocytes correlated with survival [249].

Advanced CD30 CAR-T Platforms and Combination Strategies

The Phase II trial NCT03196830 evaluated anti-CD30 CAR-T cells, with or without PD-1 blockade, in patients with R/R CD30^+^ lymphoma. The CAR incorporated a CD30-specific single-chain variable fragment (scFv), a linker, a 4-1BB costimulatory domain, and a CD3ζ signaling domain. Thirteen patients were enrolled (9 with cHL, 2 with AITL, 2 with gray zone lymphoma (GZL)); 12 patients were evaluable for response. All received fludarabine/cyclophosphamide lymphodepletion. Patients were assigned to three cohorts: cohort 1 (n = 4), 1 × 10^6^ CAR-T cells/kg; cohort 2 (n = 3), 1 × 10^7^ CAR-T cells/kg; cohort 3 (n = 5), 1 × 10^7^ CAR-T cells/kg plus anti–PD-1 therapy. Across evaluable patients, ORR was 11/12 (91.7%), comprising CR 6/12 (50.0%) and PR 5/12 (41.7%); PD occurred in 1/12 (8.3%). Response-evaluation criteria not stated. In cohort with additional anti-PD-1 antibody infusion, ORR reached 5/5 (100%) with CR 4/5 (80%); no grade ≥3 CRS occurred. Across all patients, TRAEs included CRS (33.3%; grade ≥3 in 8.3%), cytopenias, fatigue, nausea, diarrhea, and rash; no neurotoxicity was observed. The regimen demonstrated high efficacy with acceptable toxicity in heavily pretreated patients [250].

The Phase I trial NCT03602157 evaluated anti-CD30 CAR-T cells co-expressing CCR4 (CCR4.CD30 CAR T) in 12 patients (10 with R/R cHL; 2 with CD30^+^ CTCL) who had received a median of 5.5 prior lines. All patients underwent fludarabine–bendamustine lymphodepletion. The product combined an anti-CD30 CAR with CCR4 to enhance tumor trafficking. Response-evaluation criteria not stated. Among 8 evaluable cHL patients, responses were CR 6/8 (75%), including one ongoing ≥2.5 years post-infusion, and PR 2/8 (25%), yielding ORR 8/8 (100%). In CTCL, responses were SD 1/2 (50%) and PD 1/2 (50%). The overall median PFS was 5.2 months; in cHL, median PFS was not reached. TRAEs were manageable: CRS occurred in 3/12, with no neurotoxicity and no DLT. A post-infusion decrease in plasma CCL17 was observed, consistent with improved CAR-T homing to tumor sites [251].

The pilot arm of the multicenter Phase II CHARIOT trial (NCT04268706) evaluated anti-CD30 CAR-T cells in patients with R/R cHL who had received ≥3 prior lines, including chemotherapy, BV and PD-1 inhibitors. All 15 patients underwent bendamustine-fludarabine lymphodepletion followed by a single infusion of anti-CD30 CAR-T cells at 2.0–2.7 × 10^8^ cells/m²; 7 patients received a second infusion at progression. Response was assessed per the Lugano criteria. After the first infusion, ORR was 73.3% (CR 60%, PR 13.3%). Among evaluable re-treated patients (n = 5), ORR was 5/5 (100%), with 3/5 CR (60%). TRAEs occurred in all patients but the regimen was well tolerated, consisting predominantly of grade 1–2 hematologic toxicities; CRS occurred in one patient (grade 1), with no neurotoxicity [252].

The Phase I/II trial NCT04288726 evaluated anti-CD30 CAR-EBV-specific T-cells in 14 patients with R/R cHL. After cyclophosphamide-fludarabine lymphodepletion, patients received one of three dose levels. Response was assessed per Lugano criteria; 13 patients were evaluable. ORR was 9/13 (69.2%), with CR 5/13 (38.5%) and PR 4/13 (30.8%); the CR rate reached 50% at dose levels 2 and 3. TRAEs occurred in 8/14 (57.1%); the safety profile was favorable: no GvHD, reversible grade 4 cytopenias in 2/14 (14.3%), and grade 1 CRS in 4/14 (28.6%) at the highest dose; no neurotoxicity was reported [253].

#### 3.6.6 Third Generation CAR-T Clinical Studies

Clinical evaluation of third-generation CARs, combining two costimulatory domains in a single construct, has focused on whether added signaling improves proliferation, persistence, and antitumor activity relative to second-generation designs.

The open-label Phase I pilot trial (ChiCTR2100053662) evaluated sequential ASCT followed by infusion of anti-CD30 CAR-T cells in 6 patients with R/R CD30^+^ lymphoma (5 with cHL; 1 with ALCL). The CAR comprised a CD30-specific scFv, CD28 and 4-1BB costimulatory domains, and a CD3ζ signaling domain. All patients received myeloablative BEAM (carmustine, etoposide, cytarabine, melphalan) with or without fludarabine, reinfusion of CD34^+^ cells for ASCT (median 3.9 × 10^6^ cells/kg), and CAR-T cells (median 7.6 × 10^6^ cells/kg). Response was assessed per the Lugano criteria, in accordance with NCCN guidelines. ORR was 6/6 (100%), comprising CR 5/6 (83.3%) and PR 1/6 (16.7%). TRAEs included cytopenias of grade ≥3 in all patients and grade 1 CRS in 5/6 (83.3%); no ICANS occurred. Hematopoietic recovery was achieved in all cases, and no severe infections were reported during the first month. At a median follow-up of 20.4 months, all patients were alive and in remission. While limited by the small cohort, these data support the feasibility, tolerability, and promising activity of ASCT followed by anti-CD30 CAR-T cells in chemoresistant disease [254].

The Phase 0 trial (ChiCTR–OPN–16009069) evaluated a third-generation CD30-targeted CAR-T cell construct incorporating both CD28 and 4-1BB costimulatory domains, designed to accelerate activation and sustain *in vivo* persistence, in patients with R/R CD30^+^ lymphoma. Nine patients were enrolled (6 with cHL, 3 with ALCL), all heavily pretreated with chemotherapy, radiotherapy, and PD-1 inhibitors. After fludarabine–cyclophosphamide lymphodepletion, patients received 0.7–3.2 × 10^7^ CAR-T cells/kg. Response was assessed per IWG-2007 criteria. At the first post-infusion assessment, CR 7/9 (77.8%) was achieved; three patients maintained remission for >28 months. One patient had SD 1/9 (11.1%), and one died from pleural hemorrhage, possibly related to local CAR-T cell hyperactivation at a tumor site with subsequent intratumoral vessel rupture. Median relapse-free survival was 13 months. CRS occurred in 6/9 (66.7%) (grade 1–2 in four cases), with no ICANS observed. Patients with higher tumor burden exhibited larger increases in IL-6 and ferritin (≥5-fold over baseline) and experienced more severe TRAEs, including the fatal hemorrhage. CAR-T cell persistence was detectable for up to six months and appeared longer with the addition of anti–PD-1 therapy [255]. All preclinical data (Table 1) and clinical data (Table 2) are summarized below.

**Table 1:** Preclinical researches of targeted immunotherapies in Hodgkin lymphoma

Target	Type of drug	Additional terms	Research object	Results	Reference
CD30	CAR-T (1st gen.)	EBV-CTLs were taken as the T-cells for modification	autologous LCL (nHL) cells *in vitro*; HDLM-2, L-428, KM-H2 (HL) cell lines *in vitro*; xenografted L-428 cell line in SCID mice *in vivo*	First-generation CD30 CAR EBV-CTLs killed CD30+ lines (up to 58%) and induced sustained regressions while retaining antiviral function.	[220]
	CAR-T (2nd gen.)	Additional CCR4 co-expression in T-cells	HDLM-2 (HL) cell lines *in vitro*; xenografted Karpas-299 (nHL) cell line in SCID mice and xenografted HDLM-2 cell line in NOG/SCID/γc^−^/^−^ mice *in vivo*	CCR4-expressing CD30 CAR-T cells improved chemotaxis and tumor homing, yielding tumor control with 57% regressions.	[226]
		TSCM were taken as the T-cells for modification	L-540, L-428 (HL) cell lines *in vitro*; xenografted L-428 (intravenous) and L-540 (subcutaneous) cell lines in NSG mice *in vivo*	TSCM-enriched anti-CD30 CAR-T cells eradicated tumors in all models and persisted with protective memory.	[227]
		N/A	L-540, L-428 (HL) cell lines *in vitro*; xenografted L-428-EGFP-luci cell line in NCG mice *in vivo*	Humanized hHRS3 CAR-T cells achieved ≥80% lysis and extended survival with a central-memory phenotype.	[228]
	CAR-T (3rd gen.)	N/A	L-540, L-428 (HL) cell lines *in vitro*; xenografted L-428 (subcutaneous) cell line in NPG mice *in vivo*	Third-generation CD30-28BBz CAR-T cells suppressed tumors and improved survival over second-generation with greater persistence and lower exhaustion.	[238]
	Monoclonal antibody MDX-060	Gemcitabine; Etoposide; Doxorubicin	L-540, L-428, L-1236 (HL) cell lines *in vitro*	5F11 plus chemotherapy was supra-additive, halving gemcitabine IC50 and increasing apoptosis; sensitivity rose with CD30 and fell with NFκB.	[166]
		CD30 cross-linking antibody	L-540 (HL) cell line *in vitro*; xenografted L-540Cy cell line (subcutaneous and disseminated) in SCID mice *in vivo*	MDX-060 mediated ADCC and growth inhibition; in xenografts caused tumor delay with 40% complete regressions and improved survival, enhanced by cross-linking.	[165]
	Monoclonal antibody MDX-1401	Monoclonal antibody MDX-060	L-540, L-428, L-1236 (HL) cell lines *in vitro*; xenografted Karpas-299 (nHL) cell line in SCID mice *in vivo*	Nonfucosylated MDX-1401 increased ADCC and extended survival vs. MDX-060, retaining activity at low CD30 expression.	[167]
	Monoclonal antibody SGN-30	N/A	L-540, KM-H2, HDLM-2, and L-428 (HL) cell line *in vitro*; xenografted L-540Cy (HL) cell line (disseminated and subcutaneous) in SCID (C.B-17) mice *in vivo*	In SCID mice, SGN-30 (4 mg/kg) achieved 100% survival in disseminated HL and dose-dependent inhibition in subcutaneous tumors.	[159]
	Monoclonal antibody XmAb2513	cAC10-IgG1 antibody; 5F11 antibody	L-540 (HL) cell line *in vitro*	Fc-engineered XmAb2513 showed 3× potency over cAC10-IgG1 and 10× over 5F11 with higher maximal cytotoxicity.	[169]
	Oncolytic viruses MV-CD30, VSV-CD30	N/A	KM-H2, L-428, L-1236 (HL) cell lines *in vitro*; xenografted KM-H2 (HL) cell line (systemic/intratumoral and disseminated) in NSG mice *in vivo*	CD30-targeted oncolytic viruses, especially VSV-CD30, reduced viability and produced durable tumor control in xenografts.	[206]
CD30/CD137	Bispecific antibody (clone 2)	N/A	KM-H2, HDLM-2, L-428, L-1236 (HL) cell lines *in vitro*	CD30×CD137 CrossMab amplified NK-mediated killing (4×), induced apoptosis, and internalized efficiently.	[194]
CD30/CD16a	Bispecific antibody Acimtamig	Activated/expanded NK cells	Karpas-299 (nHL) cell line, activated NK cells *in vitro*; xenografted Karpas-299 (nHL) cell line in NSG mice *in vivo*	Acimtamig with activated/expanded NK cells enhanced cytokines and degranulation, maintained cytotoxicity >72 h, and reduced tumor burden.	[191]
		Adoptive NK cells (AlloNK/AB-101)	Karpas-299 (nHL) cell line, activated NK cells *in vitro*; xenografted Karpas-299/Luc (nHL) cell line in NOG-hIL-15 mice *in vivo*	Acimtamig with allogeneic NK cells (AB-101) achieved ~80% lysis and suppressed tumor growth; ~90% NK binding mAb without fratricide.	[189]
		Anti-PD-1 antibody; anti-CD137 antibody	CD30+ lymphoma cells *in vitro*; CD30+ lymphoma PDX mice model (Rag2^−^/^−^IL2Rγ^−^/^−^) with autologous PBMCs *in vivo*	Acimtamig plus anti-PD-1 or anti-CD137 raised lysis to 70% and induced tumor regressions with increased CD8+ and NK activation.	[186]
		Activated/expanded NK cells	L-428, L-1236, HDLM-2, L-540Cy (HL) cell lines *in vitro*	Acimtamig recruited NK cells to kill CD30+ cells with 3–39 pM EC50, outperforming comparators without nonspecific activation.	[185]
	Bispecific antibody HRS-3/A9	PBL, NK cells	L-540, L-428, HDLM-2 (HL) cell lines *in vitro*; HPB-ALL and L-735 (nHL) cell lines *in vitro*; xenografted L-540Cy (subcutaneous) cell line in SCID mice *in vivo*	HRS-3/A9 redirected NK cells to lyse CD30+ targets and produced complete tumor regression with durable remissions in mice.	[182]
CD30/CD3	Bispecific antibody 8D10-OKT3	T-cells	RPMI-6666, L-428 (HL) cell lines *in vitro*; xenografted HH (nHL) cell line in NSG mice *in vivo*	8D10-OKT3–armed T cells killed CD30+ targets (~80% lysis), secreted cytokines, and prolonged survival while sparing CD30− cells.	[176]
CD30/CD3	Bispecific antibody DuoBody® GEN3017	T-cells	L-428 (HL) cell line *in vitro*	DuoBody-CD3×CD30 drove strong T-cell activation and >80% killing of L-428 at subnanomolar EC50; monospecific controls were inactive.	[177]
CD30/CD64	Bispecific antibody H22×Ki-4	Monocyte-derived macrophages	L-540 (HL) cell line *in vitro*	H22×Ki-4 recruited FcγRI+ myeloid cells to mediate ADCC (51% lysis) and increased macrophage phagocytosis (>75%).	[180]
CD30	^131^I–Ki-4	^111^In–Ki-4	L-540 (HL) cell line *in vitro*; xenografted (cHL) cell line in SCID mice *in vivo*	Anti-CD30 RICs with high affinity (Kd ~20–220 nm); 24-h tumor uptake 2.6–12.3% ID/g (highest with I-131–Ki-4)	[198]
EBV antigens	EBV-CTLs (dominant-negative TGFβRII)	N/A	HDLM-2 and L-1236 (HL) cell lines *in vitro*; EBV^+^ LCLs (nHL) cell line *in vitro*; xenografted EBV^+^ LCLs (nHL) cell line in SCID mice *in vivo*	DNRII-modified EBV-CTLs resisted TGFβ, preserving proliferation and cytotoxicity with sustained antitumor effects.	[232]
CD123	CAR-T (2nd gen.)	N/A	HDLM-2, KM-H2, SUP-HD1, L-428 (HL) cell lines *in vitro*; xenografted HDLM-2 cell line in NSG mice *in vivo*	CART123 eliminated HL xenografts with durable remission and functioned despite tumor-associated macrophages.	[229]

Note: Abbreviations: ADCC—antibody-dependent cellular cytotoxicity, ADCP—antibody-dependent cellular phagocytosis, CAR-T—chimeric antigen receptor T-cells, CCR4—C-C chemokine receptor 4, CD—cluster of differentiaton, DNRII—dominant-negative TGF-β receptor II, EBV—Epstein–Barr virus, EBV-CTLs—EBV-specific cytotoxic T-lymphocytes, EC50—half maximal effective concentration, EGFP—enhanced green fluorescent protein, EGFP-Luc—enhanced green fluorescent protein–luciferase reporter, HL—Hodgkin lymphoma, IC50—half maximal inhibitory concentration, IgG1—immunoglobulin G1, IL2Rγ—interleukin-2 receptor common gamma chain (γc), LCL—lymphoblastoid cell line, mAb—monoclonal antibody, MV—measles virus, N/A—Not Applicable, NF-κB—Nuclear Factor kappa-light-chain-enhancer of activated B cells, NHL—non-Hodgkin lymphoma, NCG—NOD-SCID IL2Rγ-null mice, NOG—NOD/Shi-SCID IL2Rγ-null mice, NK—natural killer, NPG—NOD-SCID IL2Rγ-null mice, NSG—NOD SCID gamma mice, PBMCs—peripheral blood mononuclear cells, PBL—peripheral blood lymphocytes, PD-1—programmed cell death protein 1, PDX—patient-derived xenograft, SCID—severe combined immunodeficiency, TGF-β—transforming growth factor beta, TGF-βRII—transforming growth factor beta receptor II, TSCM—T memory stem cells, VSV—vesicular stomatitis virus.

**Table 2:** Clinical trials of targeted immunotherapies in Hodgkin lymphoma

Target	Type of drug	Additional terms	Clinical trials ID	Phase	Disease	Status/Results	Reference
CD30	Monoclonal antibody SGN-30	Gemcitabine; Doxorubicin; Vinorelbine tartrate	NCT00337194	II	R/R cHL (n = 38) or ALCL (n = 41)	**Completed.** No ORR; SD 28.9% (Cotswolds criteria). Favorable safety.	[164]
		N/A	NCT00051597	I/II	R/R cHL (n = 21) or CD30^+^ nHL (n = 3)	**Completed.** CR 4.2% (ALCL only); SD 19.0% (Cotswolds criteria). Favorable safety.	[142]
	Monoclonal antibody MDX-060	N/A	NCT00059995	I/II	CD30^+^ lymphomas: HL (n = 63) or ALCL (n = 7) or TCL (n = 2)	**Completed.** ORR 8%: 5.6% CR, 2.8% PR; SD 34.7% (5 pts >12 months); TRAEs (≥3 grade) 7%; MTD–NR (IWG-1999 criteria).	[141]
		Gemcitabine; Dexamethasone	NCT00284804	II	R/R cHL (N = 74)	**Completed.** Results not published.	[141]
	Monoclonal antibody MDX-1401	N/A	NCT00634452	I	CD30^+^ R/R HL (N = 12)	**Completed.** No ORR; SD 66.7% (criteria NR); 2/12 pts tumor reduction (≥40% volume); PD 33%; 10/12 pts CD30^+^ circulating cells reduction.	[168]
	Monoclonal antibody XmAb2513	N/A	NCT00606645	I	HL or ALCL (N = 23)	**Completed.** ORR 8.7% (only PR); SD 47.8%; PD 43.5% (IWG-2007 criteria). TRAE 78.3% (≥3 grade 8.7%); MTD–NR; no DLT.	[170]
CD30/ CD3	Bispecific antibody CD30biAb-AATC	Autologous T-cells; GM-CSF	NCT05544968	I	R/R CD30^+^ Hematopoietic Malignancies (N = 42)	**Not yet recruiting.**	–
	Bispecific antibody GEN3017	N/A	NCT06018129	I/IIa	R/R CD30^+^ lymphomas: cHL or TCL (N = 9)	**Terminated.** Discontinued in 2025 (portfolio decision).	–
CD30/ CD64	Bispecific antibody H22×Ki-4	N/A	N/A	I	CD30^+^ lymphomas: HL (n = 8) or AITL (n = 2)	**Completed.** ORR 40%: CR 10%, PR 30%; SD 40%; CBR 80% (CT-SPD criteria). TRAE 1–2 grade; MTD–NR.	[181]
	Bispecific antibody HRS-3/A9	N/A	N/A	I/II	R/R CD30^+^ HL (N = 15)	**Completed.** ORR 13.3%: CR 6.7%, PR 6.7%; minor response 20%; SD 13.3%; mixed response 6.7%; PD 46.7% (CT-SPD criteria). TRAE 1–2 grade; HAMA 46.2%; MTD–NR.	[151,152]
		N/A	NCT01221571	I	R/R CD30^+^ HL (N = 26)	**Completed.** ORR 11.5% (PR only); SD 50%; PD 38.5% (IWG-2007 criteria). TRAE (≥3 grade) 28.6%; 1 DLT; no TR deaths.	[187]
CD30/ CD16		Pembrolizumab	NCT02665650	Ib	R/R cHL (N = 30)	**Completed.** ORR 83%: CMR 37%, PMR 47%; PD 10% (Lugano criteria). At highest dose ORR 88%. TRAE (≥3 grade) 23%.	[188]
	Bispecific antibody Acimtamig	AlloNK (AB-101); Cyclophosphamide; Fludarabine; Interleukin-2	NCT05883449 (LuminICE-203)	II	R/R cHL (N = 10)	**Terminated.** ORR 90%: CR 60%, PR 30%; PD 10% (Lugano criteria); IR 60%; CRS 30%; no ICANS/GvHD.	[190]
		Pre-activated UCB-NK cells; Cyclophosphamide; Fludarabine	NCT04074746	I/II	Refractory CD30^+^ lymphomas: cHL (n = 37) or nHL (n = 5)	**Active, not recruiting.** In Phase II dose (1 × 10^8^ NK cells/kg): ORR 94.4%: CR 72.2%, PR 22.2%; SD 5.6%; PD 0% (criteria NR). Overall cohort ORR 92.8%: CR 66.7%. No CRS/ICANS/GvHD.	[192]
CD30	¹³¹I–Ki-4 monoclonal antibody	Unlabeled Ki-4 monoclonal antibody	N/A	I	R/R cHL (N = 22)	**Completed.** ORR 27.3%: CR 4.5%, PR 22.7% (Cotswolds); (CR 5 months; PR ≤ 6 months). Cytopenias in 33%, thrombocytopenia 33%, neutropenia 14%, anemia 5%.	[199]
		Cyclophosp hamide; Fludarabine	NCT03049449	I	CD30^+^ lymphomas: cHL (n = 20) or ALCL (n = 1)	**Completed.** ORR 43%: CR 4.8%; PR 38%; SD 52%; PD 4.8% (IWG-2007 criteria). CRS 52% (3 grade 4.8%); rash 43% (3 grade 14%); neurotoxicity 24% (grade 2). Early termination.	[244]
		Cyclophos phamide; Fludarabine; Memory-enriched T-cells	NCT04653649	I	CD30^+^ lymphomas: R/R cHL ( ) or TNHL (n = 2)	**Completed.** ORR 100% (CR 50%; PR 50%); no SD/PD (RECIL 2017 criteria). CRS 6/10 (1 grade); infections 2/10; prolonged cytopenias 2/10; no neurotox/DLT.	[245]
	CAR-T cell therapy (2nd gen.)	Cyclopho sphamide; Fludarabine; Gemcitabine; Mustargen; Nab-paclitaxel	NCT02259556	I/II	CD30^+^ lymphomas: cHL (n = 17) or ALCL (n = 1)	**Recruiting.** ORR 39% (all PR); SD 33%; PD 28%; no CR (IWG-2007 criteria). TRAE ≥ 3 grade 11%.	[246]
CD30		No lymphodepletion	NCT01316146	I	CD30^+^ lymphomas: HL (n = 6), ALCL (n = 2) or composite DLBCL/HL (n = 1)	**Withdrawn.** ORR 33.3% (all CR); SD 33.3%; PD 33.3% (IWG-2007 criteria). Minimal TRAE; no CRS/infections.	[247]
		Cyclophos phamide; Fludarabine; Bendamustine	NCT02690545	I/II	R/R cHL (N = 40)	**Recruiting.** Fludarabine-LD: ORR 72%: CR 59%, PR 13%; SD 9%, PD 19% (Lugano criteria). Bendamustine-only LD: no ORR. TRAE ≥ 3 grade (hematologic); CRS 24% (grade 1).	[197,198]
			NCT02917083	I			
		Cyclopho sphamide; Fludarabine; Anti-PD-1 antibody	NCT03196830	II	CD30^+^ lymphomas: cHL (n = 9), AITL (n = 2) or GZL (n = 2)	**Unknown status.** CAR-T only: ORR 91.7%: CR 50%, PR 41.7%; PD 8.3% (criteria NR). CAR-T + anti-PD-1: ORR 100% (CR 80%). CRS 33.3% (≥3 grade 8.3%); no neurotoxicity.	[250]
		CCR4.CD30 CAR-T; Fludarabine; Bendamustine	NCT03602157	I	CD30^+^ lymphomas: R/R cHL (n = 10) or CTCL (n = 2)	**Recruiting.** cHL: ORR 100%: CR 75%, PR 25%; CTCL: SD 50%, PD 50% (criteria NR). Median PFS 5.2 month (not reached in cHL). CRS 25%; no neurotoxicity/DLT.	[251]
		Fludarabine; Bendamustine; CAR-T re-infusion	NCT04268706	II	R/R cHL (N = 15)	**Active, not recruiting.** After 1st infusion: ORR 73.3%:CR 60%, PR 13.3% (Lugano criteria). Re-treatment (n = 5): ORR 100% (CR 60%). TRAE mostly 1–2 grade (hematologic); CRS (only 1 grade); no neurotoxicity.	[252]
		CAR-EBV-specific T cells; Cyclopho sphamide; Fludarabine	NCT04288726	I/II	R/R CD30^+^ cHL (N = 13)	**Recruiting.** ORR 69.2%: CR 38.5%, PR 30.8%; CR 50% at Dose Level 2–3 (Lugano criteria). No GvHD; reversible cytopenias (4 grade) 14.3%; CRS (1 grade) 28.6%; no neurotoxicity.	[253]
	CAR-T cell therapy (3rd gen.)	Armustine; Etoposide; Cytarabine; Melphalan; Fludarabine; ASCT	ChiCTR2100053662	I	R/R CD30^+^ lymphomas cHL (n = 5) or ALCL (n = 1)	**Recruiting.** ORR 100%: CR 83.3%, PR 16.7% (Lugano/NCCN criteria). Cytopenias (≥3 grade); CRS (grade 1) in 83.3%; all in remission at 20.4 months median.	[254]
		Cyclopho sphamide; Fludarabine; Anti-PD-1 antibody	ChiCTR-OPN-16009069	0	R/R CD30^+^ lymphomas: cHL (n = 6) or ALCL (n = 3)	**Recruiting.** ORR 77.8% (only CR); SD 11.1% (criteria NR); 1 death (pleural hemorrhage). Median RFS 13 months. CRS 66.7% (grade 1–2 in 4 cases); no ICANS.	[255]

Note: Abbreviations: AITL—angioimmunoblastic T-cell lymphoma, ALCL—anaplastic large cell lymphoma, ASCT—autologous stem cell transplantation, CBR—clinical benefit rate, cHL—classic Hodgkin lymphoma, ChiCTR—Chinese Clinical Trial Registry, CMR—complete metabolic response, CR—complete response, CRS—cytokine release syndrome, CTCL—cutaneous T-cell lymphoma, CT-SPD—computed tomography sum of product of diameters, DLBCL—diffuse large B-cell lymphoma, DLT—dose-limiting toxicity, EBV—Epstein–Barr virus, GM-CSF—granulocyte–macrophage colony-stimulating factor, GvHD—graft-vs.-host disease, GZL—gray zone lymphoma, HAMA—human anti-mouse antibody, HL—Hodgkin lymphoma, ICANS—immune effector cell–associated neurotoxicity syndrome, IR—infusion reaction, IWG—International Working Group (lymphoma response criteria), LD—lymphodepletion, MTD—maximum tolerated dose, NCCN—National Comprehensive Cancer Network, NHL—non-Hodgkin lymphoma, NK—natural killer, NR—not reported, ORR—overall response rate, PD—progressive disease, PFS—progression-free survival, PMR—partial metabolic response, PR—partial response, RECIL—Response Evaluation Criteria in Lymphoma, RFS—relapse-free survival, R/R—relapsed/refractory, SD—stable disease, TCL—T-cell lymphoma, TNHL—T-cell non-Hodgkin lymphoma, TRAE—treatment-related adverse event, UCB—umbilical cord blood.

## 4 Discussion

CD30-directed therapies have substantially expanded the treatment armamentarium for classical Hodgkin lymphoma, offering meaningful clinical benefit across diverse patient populations. Nevertheless, accumulating evidence from both trials and real-world practice indicates that their effectiveness is shaped by a range of biological and clinical constraints. These constraints arise from treatment-related toxicities, limitations in durability of response, and the influence of tumor burden and the immunosuppressive TME.

### 4.1 Limitations and Strategies for Overcoming Them

BV has demonstrated clinical efficacy in cHL, underscoring the relevance of CD30 as a therapeutic target. In clinical trials, BV achieved high response rates even in heavily pretreated patients, leading to accelerated approval and subsequent incorporation into treatment standards. However, real-world and trial experience have also revealed several limitations. First, BV monotherapy rarely induces durable remissions without additional therapy, although 5-year overall survival among patients with refractory cHL after BV exceeds 40%, the proportion who remain in complete remission without subsequent treatment is ~10% [176]. Second, cumulative toxicity, most notably peripheral neuropathy, is a significant concern. In ECHELON-1, neuropathy was the most common adverse event (67% of patients) and frequently necessitated dose reductions or treatment modifications [256]. Although neuropathy often improves or resolves over time, in some patients it persists and impairs quality of life [257]. Finally, clinical failure of BV highlights the need for alternative strategies: patients with disease refractory to both BV and PD-1 inhibitors experience poor outcomes [258]. Notably, CD30 expression typically persists at progression after BV [259], supporting the rationale to retarget CD30 using alternative modalities.

CAR-T cell therapy targeting CD30 has emerged as an innovative strategy to overcome resistance in cHL. Early clinical studies established the feasibility of this approach but also highlighted key determinants of efficacy. In trials where anti-CD30 CAR-T cells were infused without lymphodepletion, the ORR was 33.3%, with approximately one-third of patients achieving CR. Toxicity was minimal: no CRS was observed, and no meaningful immunosuppression or infectious complications were reported [247]. The modest activity in the absence of lymphodepletion mirrors experience with anti-CD19 CAR-T cells: competition from endogenous lymphopoiesis and immunosuppressive cells in the TME can limit engraftment and expansion of infused T-cells [260,261].

The addition of lymphodepleting chemotherapy (fludarabine/cyclophosphamide and bendamustine optionally) substantially increased anti-tumor activity in cHL. In a pooled analysis of two studies in heavily pretreated patients, fludarabine-containing regimens yielded an ORR of about 72% (CR 59%), whereas a small cohort treated without fludarabine showed no objective responses [248]. These findings suggest that lymphodepletion not only creates a homeostatic niche for CAR-T cell expansion but also reduces tumor burden and regulatory immune populations [262] and may improve tumor-antigen recognition by mature DCs and M1-macrophages [263]. MTV >60 mL was associated with inferior PFS after CAR-T cell therapy, whereas neither CAR-T cell expansion nor PD-1 expression on T-lymphocytes correlated with outcome [249]. These observations suggest that high tumor burden and an immunosuppressive microenvironment are major barriers, and that reducing both before CAR-T cell infusion may be beneficial.

Subsequent refinements in CD30-directed CAR-T cell design and deployment have yielded encouraging results. In a Phase II study, combining CAR-T cells with a PD-1 inhibitor produced an ORR of 91.7%; in one cohort receiving the combination, ORR reached 100% with 80% CR, without notable additional toxicities. This effect is consistent with anti-PD-1 therapy mitigating T-cell exhaustion in cHL and enhancing proliferation and cytotoxicity of redirected CAR-T cells [250]. Another strategy aims to improve tumor trafficking: CCR4 co-expression on CAR-T cells enables migration along CCL17/CCL22 gradients that typically recruit CCR4^+^ Tregs to lymphoma sites. In a Phase I study of CCR4.CD30 CAR-T cells, all evaluable patients with cHL achieved responses, including 75% CR; one patient remained in complete remission for >2.5 years, suggesting improved long-term persistence. A post-infusion decrease in plasma CCL17 levels was used as a pharmacodynamic surrogate for CAR-T cell homing and engraftment to involved lymph nodes [251]. Overall, the safety profile of anti-CD30 CAR-T cell therapy appears favorable: across studies, the reported incidence of grade 2–3 CRS ranged from 8% to 24%, and ICANS was rare to absent, distinguishing cHL from many B cell malignancies where severe CRS and ICANS are more common [264]. The relatively low abundance of HRS cells together with an immunosuppressive TME may limit exuberant activation of infused T-cells.

Nevertheless, rare severe events have been reported: in a pilot study of third-generation anti-CD30 CAR-T cells, a fatal pleural hemorrhage occurred, possibly related to localized cytokine release and vascular injury within a heavily infiltrated lesion [255]. Such cases were associated with high tumor burden and marked IL-6 and ferritin peaks, with more pronounced toxicity. These observations highlight the need for caution in patients with bulky disease; bridging debulking with chemotherapy or radiotherapy prior to CAR-T cell infusion, and/or fractionated dosing, may be warranted.

BiAbs and innate immune cell-based approaches expand the treatment options in cHL, offering operationally simpler alternatives to autologous CAR-T cell therapy. AFM13—a tetravalent biAbs that engages CD30 on tumor cells and CD16a on NK cells, is a leading example. Unlike T-cell–redirecting agents, harnessing innate effectors has been associated with low rates of CRS and GvHD [265]. In a recent Phase I study combining AFM13 with allogeneic NK cells in patients with R/R cHL who had received a median of seven prior lines of therapy (all refractory to BV and PD-1 inhibitors), ORR was 92.8% with 66.7% CR. Notably, AFM13-NK therapy showed no CRS, ICANS, or GvHD; a single grade 2 infusion reaction was reported, indicating favorable tolerability. The limited *in vivo* persistence of donor NK cells (typically detectable in blood for about 3 weeks) may contribute to shorter remission durations; accordingly, this approach is being explored as a bridge to transplant or with repeat courses [192,258].

The concept of arming effector cells with biAbs has demonstrated efficacy and is advancing rapidly. In parallel with NK-directed constructs, bispecifics that redirect T-lymphocytes are being developed. Notably, CD30×CD3 biAbs can promote formation of an immunological synapse between cytotoxic T-cells and HRS cells. One approach uses *ex vivo* arming: a patient’s autologous T-lymphocytes are activated and loaded with a bispecific reagent and then reinfused [176]. This product requires no genetic modification yet transiently confers dual specificity. Importantly, the antibodies used in this setting recognize a non-overlapping CD30 epitope relative to the chimeric antibody AC-10 (the BV backbone). This feature could help circumvent resistance mechanisms such as neutralization by soluble CD30 or competition with prior anti-CD30 antibodies and may enable re-targeting of CD30 in BV-refractory disease.

### 4.2 Prospective Therapeutic Pathways

The success of immune approaches is shaped by the properties of the TME and by optimal deployment strategies. cHL features a strongly immunosuppressive TME: despite abundant immune infiltrates, a PD-1^high^ niche of exhausted T-lymphocytes and M2-macrophages expressing PD-L1 forms around HRS cells [68]. HRS cells actively suppress antitumor effectors via PD-L1 expression, secretion of TGF-β and galectin-1, and additional mechanisms [266]. CD4^+^ T-cells, which form «rosettes» around HRS, lose cytotoxic function under chronic stimulation by PD-1 ligands and tumor-derived cytokines [68,92]. CD8^+^ T-cells undergo apoptosis through FasL–Fas signaling emanating from the tumor, and NK cells are functionally suppressed by PD-1/PD-L1 interactions and other local immunoregulatory factors [85,97]. CCR4^+^ Tregs are actively recruited by HRS-derived CCL17 and CCL22, further reinforcing immunosuppression [99]. Collectively, these features help explain why, in the absence of adjunctive interventions, neither endogenous nor infused effector cells fully eradicate HRS cells. Current strategies aim to disrupt this tolerance: PD-1 blockade can reinvigorate exhausted T-cells [267] and has shown favorable activity in combination with targeted agents, illustrated for CAR-T cells and AFM13 [188,250]. Co-expression of CCR4 on CAR-T cells promotes homing to tumor sites by following CCL17/CCL22 gradients, helping them navigate Treg-recruiting chemokine niches [226,251]. In addition, these modalities can actively remodel the TME. For example, lysis of HRS cells by CAR-T cells or AFM13 releases tumor antigens and pro-inflammatory signals that recruit additional effector cells [268]. In preclinical studies, combining biAbs with immunostimulants increased infiltration by CD8^+^ T-cells and NK cells, boosted local IFN-γ, TNF-α, and IL-2 production, and achieved complete tumor regression [186]. Together, these observations suggest that combinatorial immunotherapy may overcome established barriers and promote eradication of residual HRS cells that escape single-agent approaches.

Another key issue is sequencing and combination with existing treatments. Because BV is now commonly used upfront in cHL, relapsed tumors may be selected under prior anti-CD30 pressure [269]. However, the frequent persistence of CD30 at relapse supports continued vulnerability to CAR-T or bispecific immunotherapeutics [259]. Conversely, prior PD-1 blockade may favor subsequent cell therapy; deep, durable remissions have been reported when CAR-T cells were administered shortly after nivolumab [250]. We hypothesize that high-dose chemotherapy with ASCT similarly creates a transient therapeutic window for CAR-T cell therapy: lymphodepletion and tumor debulking reduce immunosuppressive constraints within the microenvironment, while autologous stem-cell rescue restores hematopoiesis [61]. Deploying CAR-T cells in this setting may eradicate minimal residual disease that might otherwise progress over time [270]. Early experience with this sequential strategy is encouraging: in a pilot study of refractory patients post-ASCT, a single infusion of third-generation anti-CD30 CAR-T cells produced 100% responses (83% CR) with acceptable toxicity; all patients remained in remission at a median follow-up of 20 months [254]. These observations, although based on a small cohort, support the hypothesis that earlier use of CAR-T cell therapy, e.g., immediately after transplantation or as consolidation in high-risk patients, may be more effective than treatment administered after multiple prior lines of chemo-immunotherapy. Similarly, for AFM13 and other emerging agents, bridging therapy prior to transplantation is being explored, and in selected patients for whom transplantation is contraindicated, these approaches may be considered as potential alternatives.

Finally, the long-term durability of immune control remains an open question. Although CD30-directed therapies can induce complete remissions, some patients relapse, motivating evaluation of maintenance strategies. For CAR-T cells, limited persistence of functional memory remains a central challenge [271]. Unlike anti-CD19 CAR-T therapy in leukemia, where normal B-lymphocytes provide ongoing low-level antigen exposure that may support persistence, in cHL the targeted cells are sparse and can disappear entirely with successful therapy [244,272]. Potential approaches include re-dosing (e.g., repeat CAR-T cell infusion at signs of progression, as explored in CHARIOT trial), maintenance with low-intensity anti-CD30 agents, and combination with PD-1 blockade [252]. PD-1 inhibition may prolong CAR-T cell persistence: in the study by Wang et al., third-generation anti-CD30 CAR-T cells persisted for up to 6 months and longer with subsequent PD-1 blockade [255]. For AFM13-NK, repeat courses or maintenance infusions of AFM13 may extend remissions; in some cases, AFM13-NK has served as a bridge to ASCT [8]. The risk of antigen escape, defined as the emergence of tumor variants with reduced or absent CD30 expression under therapeutic pressure, warrants separate consideration. Available evidence indicates that such events are rare in cHL; even after multiple relapses, tumor cells typically retain the HRS phenotype, including CD30 [273]. Nevertheless, over the longer term it is rational to pursue multi-specific CAR-T cell strategies that target more than one antigen (e.g., CD30 together with other HRS markers such as CD123) to mitigate the risk of immune escape.

## 5 Conclusions

Thus, CD30-directed therapy in cHL has progressed from the first successful monoclonal antibodies to a broad array of advanced immunotherapeutics. BV remains a cornerstone of targeted therapy in cHL and has improved patient survival, yet its toxicity has catalyzed the search for alternative approaches. CD30-directed CAR-T cells have already induced remissions in chemotherapy-refractory settings and continue to evolve toward greater durability and efficacy. Bispecific constructs, used with allogeneic NK cells or autologous T-cells, offer a path to more broadly accessible, off-the-shelf–like immunotherapy that does not require bespoke manufacturing.

Key near-term priorities are to optimize deployment (including timing and sequencing), refine risk management, and design rational combinations with each other and with conventional modalities. Equally important is continued investigation of HRS biology and tumor-immune interactions to identify new targets and to improve existing strategies. Integrating clinical evidence with mechanistic insights into the TME and immune regulation will be essential to realize the full potential of CD30-directed therapy and to advance treatment for even the most refractory forms of cHL.

## Data Availability

Not applicable.
